# The Mechanisms of Thin Filament Assembly and Length Regulation in Muscles

**DOI:** 10.3390/ijms23105306

**Published:** 2022-05-10

**Authors:** Szilárd Szikora, Péter Görög, József Mihály

**Affiliations:** 1 Institute of Genetics, Biological Research Centre, H-6726 Szeged, Hungary; gorog.peter@brc.hu; 2 Doctoral School of Multidisciplinary Medical Science, Faculty of Medicine, University of Szeged, H-6725 Szeged, Hungary; 3 Department of Genetics, University of Szeged, H-6726 Szeged, Hungary

**Keywords:** actin, thin filament, sarcomere, length regulation, myopathy

## Abstract

The actin containing tropomyosin and troponin decorated thin filaments form one of the crucial components of the contractile apparatus in muscles. The thin filaments are organized into densely packed lattices interdigitated with myosin-based thick filaments. The crossbridge interactions between these myofilaments drive muscle contraction, and the degree of myofilament overlap is a key factor of contractile force determination. As such, the optimal length of the thin filaments is critical for efficient activity, therefore, this parameter is precisely controlled according to the workload of a given muscle. Thin filament length is thought to be regulated by two major, but only partially understood mechanisms: it is set by (i) factors that mediate the assembly of filaments from monomers and catalyze their elongation, and (ii) by factors that specify their length and uniformity. Mutations affecting these factors can alter the length of thin filaments, and in human cases, many of them are linked to debilitating diseases such as nemaline myopathy and dilated cardiomyopathy.

## 1. Introduction

Muscle contraction relies on the precise arrangement of myofibrils, composed of serially connected contractile units called sarcomeres. Sarcomeres are filamentous structures comprised of actin-based thin filaments and myosin-based thick filaments that slide along each other to generate force, ultimately leading to muscle contraction [1]. The force output of a muscle can be quantified as a function of myofilament overlap and is often characterized by the sarcomere length–tension relationship [2,3]. The length of the thick filaments is constant (~1.65 μm) and is conserved across vertebrate species and muscle types. However, the length of the thin filaments is variable, implying that thin filament length determines the extent of the overlap between the myofilaments, which is one of the important determinants of the amount of force generated. Accordingly, thin filament length correlates well with the physiological requirements of the muscle as slow-twitch fibers have longer thin filaments, while fast-twitch fibers have shorter thin filaments in general [4,5,6,7]. This observation also suggests that slow-twitch muscles have longer optimal sarcomere resting lengths, which might be one reason as to why they are able to efficiently maintain longer-term contractions. Therefore, to achieve optimal contraction characteristics, the thin filament length is expected to be precisely specified.

This review summarizes the current understanding of how thin filament length is regulated. Although significant discoveries in the field have been made in *C. elegans* (for reviews see: [8,9]), our review primarily focuses on vertebrates and *Drosophila*. We highlight the most important factors that control the assembly and elongation of thin filaments, and discuss the most prominent ideas proposed to explain how thin filament length and uniformity are achieved. Because impairing the function of many of the proteins reviewed here leads to the development of muscle diseases, the mechanisms of thin filament assembly and length regulation are of important medical relevance, which have recently been reviewed in several excellent papers [10,11,12]. For this reason, and due to the space constraint, the focus of this review will be on model system studies that have already provided us with a wealth of fascinating data.

## 2. Thin Filament Structure

**Figure 1:**
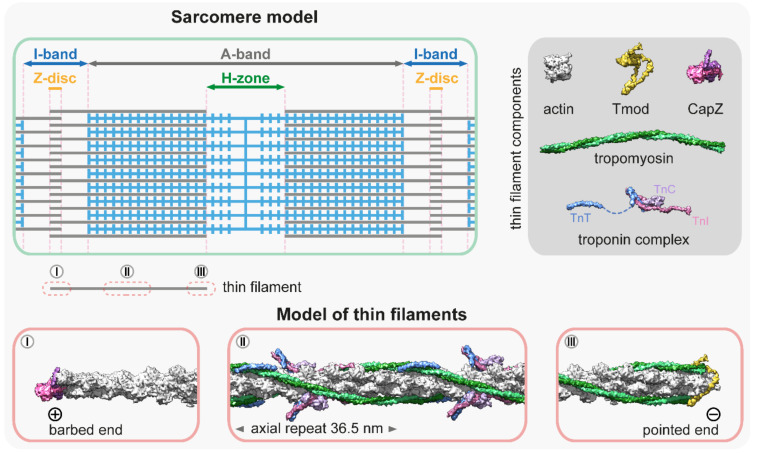
**Molecular representation of the thin filaments.** The schematic at the top left is a simplified sarcomere model with thick (blue) and thin filaments (gray), and the major sarcomeric regions. The dark gray box at the top right shows the main components of the thin filaments [13,14,32,33,34,35]—PDB: 5JLF, 4PKI, 4PKG, 7PDZ, 1C1G, 4Y99, 1J1E, 2Z5H. The molecular models below depict three representative thin filament regions: (**I**) The thin filament proximal to the barbed end is not decorated with tropomyosin and troponin, however, it is associated with other factors (elastic filaments, nebulin, Z-disc proteins, etc.) that are not depicted in this representation. The barbed end is capped with a CapZ heterodimer [34]; PDB: 7PDZ. (**II**) The central region is associated with tropomyosin–troponin complexes [25]; PDB: 7KO4. This section depicts a full axial repeat. Note that in some muscles, this part of the thin filament also contains two nebulin molecules. (**III**) The pointed end is capped by tropomodulin (Tmod) [33]; PDB: 4PKI, 4PKG. The association of Tmod to the pointed end is significantly enhanced by tropomyosin binding.

All muscles contain thin filaments (diameter of 6–10 nm) that consist of actin, tropomyosin (Tm), and troponin (Tn) in a 7:1:1 stoichiometry (Figure 1). Upon neuronal activation, Ca^2+^ is released from the sarcoplasmic reticulum, and triggers a conformational change in Tn, which shifts the azimuthal position of Tm on F-actin to allow for actin–myosin interactions. Over the past years, we have gained a good structural understanding of the thin filaments [13,14,15,16] that were analyzed by various advanced methods including X-ray fiber diffraction, electron microscopy (EM), and more recently, by cryo-EM [17,18,19,20,21,22,23,24,25]. Hence, the current models provide a highly resolved structure to provide insights into many aspects of their function and regulation (Figure 1). The structural core of the thin filament is the helical F-actin polymer that repeats once every 14 monomers and has an average axial repeat size of 36.5 nm (Figure 1). This structure is evolutionary highly conserved, although slight variations have also been reported. For instance, in the insect flight muscle, the average axial thin filament repeat size is 38.7 nm [26,27,28]. As much as the actin filaments themselves, the thin filaments are polarized structures with their barbed ends crosslinked in the Z-disc and capped by CapZ, and have their pointed ends extending toward the M-line where they are capped by tropomodulin (Tmod) (Figure 1). The thin filaments are stabilized by Tm molecules [29]; notably there are two pairs of Tm in every axial repeat, one on each side of the actin filament. The Tm dimers are linked end-to-end to form continuous α-helical coiled-coils that follow the F-actin long-pitch helix, where they regulate muscle contraction by sterically blocking the myosin target sites on the thin filament (Figure 1) [30]. Each Tm dimer has a regulatory heterotrimeric troponin complex attached to it, composed of troponin T (TnT), troponin C (TnC), and troponin I (TnI). TnT is the Tm binding subunit; TnC is the Ca^2+^-binding regulatory subunit; and TnI is the inhibitory subunit (Figure 1) (for a recent review see: [31]).

Conventional structural reconstructions provide an averaged image of the thin filaments, however, it has long been known that thin filaments are not perfectly uniform [36,37]. It has been shown that the terminal units of the actin filaments adopt a conformation that is different to the rest of the filament [38,39]. Moreover, actin-binding proteins can exert conformational changes on the filaments (i.e., formin binding to the barbed end can induce a more flexible conformation through long-range allosteric effects [40,41]; and ADF/cofilins binding near the pointed end can shorten the helical pitch of the filaments [42,43,44]). Therefore, the structure of the ends of the thin filaments is likely to differ from the averaged reconstructions, and it might provide us with essential cues to better understand the mechanisms of filament length regulation.

In situ cryo-electron tomography and cryo-focused ion-beam scanning electron microscopy analysis allow for the molecular and structural analysis of thin filaments in their native environment and have been proven to be powerful enough to explore the local structural differences at the single sarcomere and even at the single thin filament levels [45,46]. For example, it was revealed that the position of Tm differs in the I-band from that in the A-band, where the binding of myosin to the thin filament shifts Tm from the C-state to the M-state. Tm in the I-band remains in the C-state and the transition happens in one Tm unit immediately after the A-band/I-band transition [46]. Nevertheless, the use of these techniques has remained limited in regions of the Z-disc and the M-band/H-zone, where a large number of regulatory and structural proteins are present, forming pleomorphic densities on the EM images, thereby prohibiting the identification of individual proteins and precise reconstruction of the filament ends. One alternative to resolve the composition and arrangement of these dense sarcomeric regions can be fluorescent nanoscopy, as was recently demonstrated [47,48]. Single-molecule localization microscopy combined with structure averaging allows for the determination of the position of sarcomeric proteins with a precision of <10 nm, and it has been successfully used to reconstruct various large protein complexes (for a recent review see [49,50]). Future studies using the combination of these new methods will hopefully provide us with superior molecular models of the pointed and barbed ends of the thin filaments, which is essential to a better understanding of their dynamics and length regulation.

## 3. Thin Filament Assembly

**Figure 2:**
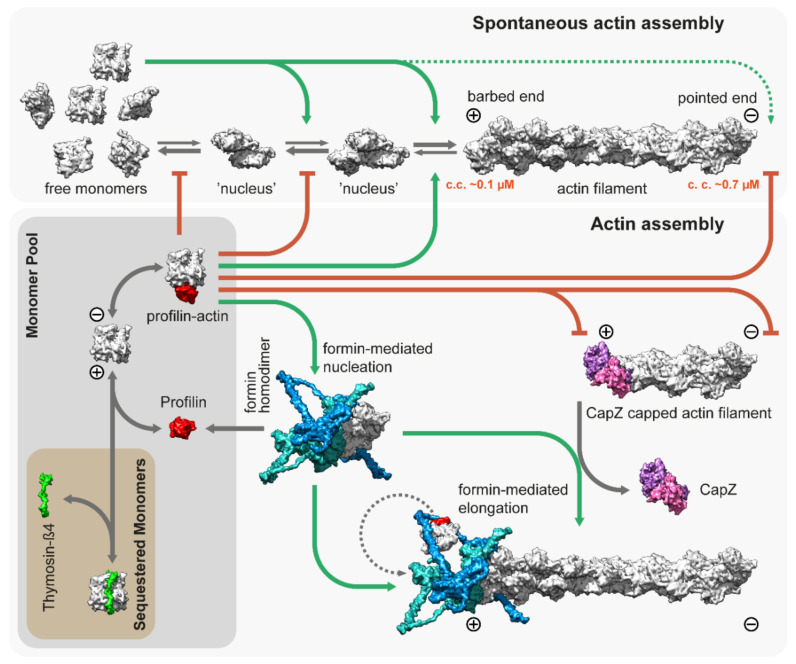
**Spontaneous and regulated assembly of actin filaments.** Free G-actin monomers can form nucleation seeds, which can elongate rapidly from both their barbed and pointed ends, although the critical concentration is significantly lower for the barbed ends (~0.1 µM). Thymosin-β4 sequesters actin monomers and prohibits uncontrolled actin assembly in vivo. Similarly, profilin [53]; PDB: 2BTF, bound to actin monomers, inhibits both spontaneous nucleation and growth from the pointed end. However, it allows for barbed end association and feeds actin monomers to formins, which are actin nucleation and elongation factors. The figure depicts the template-based [54,55] structure of DAAM1 using multiple PDB models [56,57,58]; PDB: 2Z6E, 2V8F, 1Y64. CapZ bound to the filaments prevents growth from the barbed end [34]; PDB: 7PDZ.

The backbone of the thin filament is an F-actin molecule, which is assembled from a G-actin monomer pool. Actin monomers polymerize spontaneously under physiological salt conditions starting with a slow nucleation step, in which a few actin monomers combine to form a nucleus or ‘seed’ for subsequent elongation (Figure 2). The association of the monomers is fast, but the dimers and trimers are very unstable. The nucleation or ‘seed’ formation step is followed by elongation in which monomers bind to (and dissociate from) the two ends of the filaments. Elongation has different characteristics on the opposite ends of the filament as barbed ends grow much faster than pointed ends with a significantly lower “critical concentration” (~0.1 µM and ~0.7 µM, respectively), and elongation is favored when the concentration of free G-actin exceeds the critical concentration (Figure 2) [51,52].

Actin is an abundant protein with a concentration well above the critical concentration in non-muscle cells, ranging from 50 to 200 µM [59,60]. In embryonic muscle cells, almost half of the actin pool is in a monomeric form, but the G-actin concentration decreases significantly during development; in adult muscles, it is just above the critical concentration (~0.2–0.3 µM) for barbed ends [61,62]. In addition, most of the actin monomers are bound either to profilin or thymosin-β4, which shields the barbed end side of actin monomers and prevents spontaneous nucleation [53,63,64]. While thymosin-β4 completely sequesters G-actin, thereby preventing both nucleation and elongation, profilin only inhibits nucleation and pointed end elongation. Profilin–actin complexes can bind to the exposed filament barbed ends and following its incorporation, profilin dissociates from the actin protomer (Figure 2). Muscle tissues express both profilin and thymosin-β4 [65,66], however, their role in thin filament assembly and elongation has mostly been unexplored. Nevertheless, one study demonstrated that overexpression of profilin resulted in thin filament and sarcomere elongation in the flight muscles of *Drosophila* [67], suggesting a promoting role in thin filament formation. Since nucleation is the rate limiting step of actin assembly, cells use nucleation factors to form and stabilize polymerization nuclei. While the branched actin filaments are nucleated by the Arp2/3 complex, the unbranched filaments such as the thin filaments are initiated by formins. It is possible that the WASP-homology 2 (WH2) domain containing “nucleators” (such as Spire, Cordon-bleu, or Leiomodin) also play a role in thin filament assembly, although it is unlikely that they could mediate this process without formins [68]. Formins are highly conserved, multidomain proteins characterized by the presence of two formin homology domains (FH1 and FH2) [69]. The FH2 domains can directly bind G- and F-actin and are able to nucleate actin filaments by stabilizing actin dimers [70], thus overcoming the kinetic barrier of nucleation (Figure 2). After nucleation, formins can remain bound to the barbed end of the filaments and promote their processive elongation [71,72]. This “processive capping” allows formins to protect the growing barbed end from the inhibitory effects of capping proteins such as CapZ (Figure 2) [73,74,75]. The flexible FH1 domains can interact with profilin–actin complexes and provide new subunits to the growing filament [76,77,78,79]. Additionally, formins possess other activities including the ability to bind along the lengths of actin filaments or microtubules, promoting bundling and crosslinking or coordination [80,81,82,83,84,85,86,87].

Eukaryotic species have multiple formin proteins and it has been shown that in vertebrates, 13 out of the 15 formin genes are expressed during postnatal heart development [88], while in the *Drosophila* flight muscle, all of the six fly formins are expressed at some point of development [89]. Among the sarcomeric formins, members of the FHOD family have been the most extensively studied. While FHOD1 is expressed in both the muscle and non-muscle cells, in cardiomyocytes, FHOD1 primarily localizes to the costameres and the intercalated discs, but it is largely excluded from the sarcomeres [90,91]. In contrast, FHOD3 is abundantly expressed in the heart muscle and it displays a mainly sarcomeric accumulation [92]. Silencing of FHOD3 leads to the disruption of myofibrils in cultured cardiomyocytes [93,94], and consistently, FHOD3 deficient mice die prenatally due to failed myocardial development [95]. Detailed analysis revealed that these mutant cardiomyocytes are able to form premyofibrils, however, they fail to mature into myofibrils [95]. In humans, FHOD3 mutations have been associated with hypertrophic and dilated cardiomyopathies [96,97,98,99]. In addition, FHOD3 has been shown to play a maintenance role in the adult mouse heart [100]. Similarly, muscle specific silencing of Fhos, the single *Drosophila* FHOD orthologue, severely disrupts the organization of the flight muscle myofibrils, while allowing for the proper initiation of muscle fiber development [101]. Silencing of Fhos in a later phase of flight muscle myofibrillogenesis resulted in sarcomeres with significantly narrower widths, suggesting that Fhos is necessary for the peripheral (radial) growth of the thin filaments. Despite these advances, the molecular mechanisms of FHOD type formins remain largely obscured. In bulk polymerization assays using sarcomeric actin, both FHOD1 and FHOD3 were unable to nucleate actin filaments [93,102], although it was later demonstrated that FHOD1 can nucleate actin filaments from cytoplasmic actin [103]. Furthermore, the findings on the localization of FHOD3 in myofibrils is also somewhat controversial. Most studies have shown that FHOD3 is localized to broad stripes in the A-band, more precisely, in the C-zone [93,94,95,100,104,105], and it was demonstrated that this localization is dependent on a direct interaction with the thick filament associated MyBP-C protein [105]. However, a FHOD3 enrichment has also been detected at the Z-discs in the mouse and human adult heart sections and in the extracted myofibrils [88,94]. In contrast to FHOD, *Drosophila* Fhos is able to nucleate both cytoplasmic and sarcomeric actin, it allows elongation in the presence of profilin, and it protects the barbed ends from capping protein binding [103]. Accordingly, Fhos is localized to the Z-disc [101] into the immediate vicinity of the barbed ends, as suggested by nanoscopic analysis [47]. Surprisingly, however, flies with a mutation abolishing the barbed end binding and nucleation activity of Fhos have small (short and thin) but properly organized sarcomeres [101]. Based on these observations, the common feature of FHOD type formins is that they are not required for the synthesis of the initial pool of sarcomeric thin filaments. Furthermore, their sarcomeric localization and biochemical activities suggest that their barbed end binding activities are only secondary or negligible, and therefore FHOD type formins may primarily use their actin side binding and bundling activity to organize pre-existing filaments into ordered thin filament arrays during myofibrillogenesis. Nevertheless, FHOD dependent actin assembly is required to achieve the mature sarcomere size.

The other well-established sarcomeric formin is DAAM. DAAM1 is highly expressed in the developing heart of mice, and DAAM1 deficient animals display multiple cardiac defects and die during embryonic development [106]. In humans, single copy-number deletion of DAAM1 leads to congenital heart defects [107]. DAAM2 is also expressed in a smaller amount in the heart and the double KO animals exhibit more severe phenotypes, suggesting partially redundant functions [108]. In-depth analysis revealed that in the absence of DAAM1, the F-actin content of cardiomyocytes was significantly decreased and the remaining thin filaments were disorganized. The recognizable sarcomeres were considerably shorter and more sparsely distributed than the wild-type sarcomeres [106]. Muscle specific silencing of DAAM in *Drosophila* resulted in similar complex defects in sarcomere organization with reduced thin filament level and disrupted the Z-disc and M-band organization, affecting both the heart and somatic muscles. In less affected animals, the sarcomeres were recognizable, however, they were significantly shorter and narrower [109]. Regarding their biochemical activities, it was demonstrated that both vertebrate and *Drosophila* DAAM are able to nucleate and processively elongate actin filaments from profilin–actin. Furthermore, similarly to FHOD, DAAM is also able to bind to the sides of the actin filaments [75,85,110,111]. These findings suggest that DAAM is directly involved in the polymerization of sarcomeric actin filaments during the early steps of myofibrillogenesis. These activities presume barbed end localization and accordingly, DAAM1 is localized to Z-bands in the primary cultured cardiomyocytes and mature cardiomyofibrils isolated from mice [88]. However, in the skeletal muscles, DAAM1 accumulates either into wider stripes in the A-band (most likely in the C-zone) or in the I-band along the sides of the Z-discs [109]. The subcellular localization of DAAM in *Drosophila* is also quite intricate as in the flight muscle, it was detected in a narrow stripe in the middle of the H-zone, in a seemingly F-actin-free area, and also in the Z-disc, where nanoscopic analysis suggests a barbed end association [47,109]. In developing larval body wall muscles, the DAAM staining resolves into two bands along the M-line, whereas in fully matured larval body wall muscles, the DAAM staining relocates to a region flanking the Z-disc [109].

Recently, a third formin, Dia, has also been implicated in myofibrillogenesis in *Drosophila* by regulating the length and width of each sarcomere during flight muscle development [112]. Besides myofibrillogenesis, Dia is also required during myoblast fusion to form actin foci by regulating actin nucleation directly and through the Arp2/3 complex [113] Similarly, the Dia orthologue CYK-1 is a regulator of lattice growth and maintenance in striated muscles of *C. elegans* [114].

Taken together, mounting evidence suggests that from worms to humans, the thin filament arrays are assembled, organized, and maintained by the activity of multiple formins, which is in harmony with the highly complex nature of the sarcomeres and with the known variability of the muscle types. Whereas some formins appear to provide specific muscle functions, depletion of the formins individually failed to completely abolish the formation of sarcomeric thin filaments, indicating a significant level of redundancy. Therefore, appropriately designed double mutant analysis might be important to better understand the role of formins during myofibrillogenesis. In addition, we note that although subcellular localization could be an important indicator of a molecular function in general, interpretation of the current localization data for formins appears rather challenging as the available information is often contradictory and/or inconsistent with the biochemical data. The apparent discrepancies are most likely caused by multiple factors. Since many antibodies target the most conserved FH2 domain, it is possible that the existing antibodies are not specific enough, and in a tissue where multiple formins are expressed, their staining pattern could be misleading. Furthermore, it became evident that the localization pattern of the sarcomeric formins depends on the developmental stage, which is in line with the possibility that at least some formins exert different molecular activities during the consecutive phases of myofibrillogenesis. Finally, the localization of formins might vary in different species and/or muscle tissues. Clearly, comprehensive studies have to consider this phenomenon more carefully, and it remains a challenge to further explore the molecular mechanisms of formins during the different phases of sarcomerogenesis and to fully reconcile them with the localization pattern.

## 4. Thin Filament Elongation

After their initial assembly, thin filaments must elongate to achieve their well-defined length, fitting best to the workload of a given muscle type [115]. In addition, they need to be able to elongate in response to mechanical strain to achieve the optimal efficiency, as observed in mature cardiac muscles [116]. Fluorescently labeled actin monomer incorporation studies in chick cardiac myocytes revealed that in muscle cells, both ends of the thin filaments are dynamic, however, the subunit exchange at the pointed end is significantly higher [117]. Interestingly, the blocking of barbed end dynamics did not arrest actin filament elongation, while the blocking of pointed end dynamics was sufficient to prevent filament growth in multiple model systems [117,118]. Furthermore, it was also demonstrated that labeled Tm also incorporates at the pointed end in rat cardiac myocytes [119]. Therefore, it was concluded that, in contrast to non-muscle cells, actin filaments somehow elongate from their pointed end during myofibrillogenesis [117,118].

The thin filaments are thought to be capped at both ends; the barbed end is associated with the capping protein (CapZ) complex, and the associated regulatory factors maintain the dynamics of monomer exchange [120], while the pointed end is capped by Tmod (Figure 1). Tmods (Tmod1–Tmod4 in vertebrates) are conserved proteins that have a distinct role in length regulation of the thin filaments [121,122,123]. A single Tmod molecule interacts with the thin filament via two actin binding sites (ABS1 and ABS2) and two Tm binding sites. Tropomyosin binding enhances its pointed end capping activity by 5- to 10-fold, and once Tmod is fully bound, it blocks the addition of actin monomers to the pointed end and prevents filament elongation as well as depolymerization [33,124]. Consistently, the silencing of Tmod or impairing of its capping activity leads to thin filament lengthening in various muscle tissues [123,125,126,127]. Conversely, the overexpression of Tmod inhibits actin incorporation at the pointed end, leading to thin filament shortening. Hence, thin filament length has an inverse proportion to the expression level of Tmod, as shown in the sarcomeres of cardiac myocytes and *Drosophila* flight muscles [117,118]. However, Tmod binding to the pointed end is dynamic, and it was initially suggested that Tmod allows the non-catalyzed addition of G-actin to the pointed end by acting as a leaky cap, leading to filament elongation (for a review, see [128]. In this scenario, thin filament elongation would be regulated exclusively by the expression level of Tmod.

Independent of its precise mode of action, Tmod is considered as a negative regulator of actin elongation, whereas members of the highly homologous Leiomodin (Lmod) protein family were discovered as promoters of thin filament elongation [129,130]. The Lmod family consists of three isoforms in vertebrates (Lmod1–Lmod3), which are expressed primarily in the muscle cells and behave as strong actin nucleators in biochemical assays [129,131,132]. Lmod2 and 3 are both expressed in striated muscles with Lmod2 being the predominant isoform in cardiac muscles and Lmod3 the major isoform in skeletal muscles. Defective Lmod2 gene expression leads to dilated cardiomyopathy both in mice and humans [115,133,134], while the Lmod3 gene is associated with nemaline myopathy [131,135]. Loss of Lmod1 impairs smooth muscle cytocontractility and causes megacystis microcolon intestinal hypoperistalsis syndrome (MMIHS) in both humans and mice [136]. Initially, Lmods were proposed to be the long-sought-after muscle actin nucleators [129], however, loss of function studies and the developmental timing of their expression revealed that Lmods are unlikely to be required during the initial phases of thin filament assembly [137]. Overexpression of Lmod2 leads to the elongation of thin filaments [130,138], and its loss results in thin filament shortening [115,139]. Hence, it was proposed that Lmod2 is most likely to be involved in thin filament elongation and/or maintenance. While Lmods are essential factors in vertebrate muscles, they are not present in *Drosophila*. Curiously, a nonrelated protein, called sarcomere length short (SALS)-, displaying a number of functionally similar properties, has been found in *Drosophila*. Like Lmods in vertebrates, SALS promotes thin filament elongation and sarcomere growth in primary *Drosophila* muscle cell cultures and in the flight muscles of *Drosophila* [101,140]. Loss of SALS causes shortening of the thin filaments and lack of actin incorporation at their pointed ends. The effect caused by Tmod overexpression was also increased by the loss of SALS, which suggests that Tmod and SALS antagonize each other functionally at the filament pointed ends during thin filament elongation [101,140].

**Figure 3:**
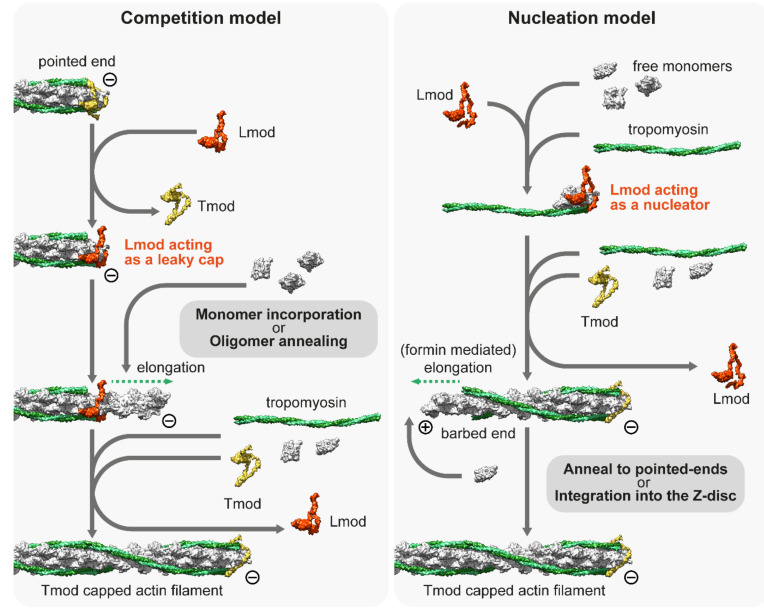
**The competition and nucleation models of Leiomodin (Lmod).** The competition model suggests that Lmod competes with Tmod, and once it is bound to the pointed end, it acts as a leaky cap, allowing the addition of monomers or short oligomers to the pointed end. The nucleation model suggests that Lmod is not able to bind to the pointed end but can act as a nucleation factor by binding at least three actin monomers and a tropomyosin dimer. The model proposes that Lmod dissociates from the actin nucleus when it begins to elongate, which can be mediated by formins. The newly formed filament can be incorporated into the Z-disc or annealed to free pointed ends. Note that both models presume that free monomers are available. The molecular model of Lmod is based on the tentative structure proposed by Tolkatchev et al. [138].

Despite the role of Lmods having been extensively studied over the last decade, no consensus has been reached on their exact molecular mechanisms; instead, two prominent but contradicting models have emerged to explain the in vivo and in vitro observations [128,141]. The ‘competition model’ suggests that thin filament elongation is regulated by the interplay between the pointed end capping Tmod and its functional antagonist Lmod, which can displace Tmod from the pointed end and promote catalyzed or non-catalyzed subunit addition (Figure 3). As an alternative, the ‘nucleation model’ suggests that Lmod nucleates new filaments, which can then anneal to the pointed ends of existing filaments or integrate into the sarcomeres during myofibril maturation and/or during repair (Figure 3). It has also been suggested that Lmod (and SALS) could cooperate with muscle-specific formins, which would mediate the processive elongation of these newly nucleated ‘mini filaments’ [109]. Part of the controversy surrounding the molecular functions of Lmods is from the fact that due to technical difficulties, the current structural and biochemical data are obtained by using Lmod fragments (instead of the full length protein) interacting with actin monomers and Tm. Results obtained from these experiments are difficult to interpret since truncated protein fragments are often insufficient to reconstitute the biological function of a full-length protein. In spite of this limitation, previous studies have exploited the modular organization of Lmods to assign functions to every domain and to decipher the function of the native protein. Lmods share several domains with Tmods, but they also have a C-terminal WH2 and a proline-rich domain (PRD) containing the extension. Lmods have only one Tm binding site, and NMR spectral analysis suggests that it folds into an α-helical hairpin, which is positioned over the N-terminus of Tm [138]. Since unobstructed Tm N-termini are only available at the pointed ends, it was interpreted as evidence supporting the “competition model”. However, a free Tm N-terminus should also be available during thin filament nucleation, therefore, it does not refute the “nucleation model”. In any case, Tm binding was shown to be important in vivo as the reduced binding affinity of this motif blocked the thin filament elongation effect of exogenous Lmod2 in cardiomyocytes [138]. ABS1 is essential for pointed end capping in Tmods and its sequence is also relatively well conserved in Lmods. However, it is controversial whether it has an actin binding activity in Lmods [132,138,142]. ABS2 is structurally similar in Tmods and Lmods, however, they appear to fulfill different functions. In Lmod, the ABS2 domain lacks the DNase I-binding loop, which is important for pointed end capping, and the ABS2 in Lmod can bind two or three actin subunits on its own, making it primarily responsible for the nucleation activity of Lmod [132]. Lmods also contain a PRD, which is an ideal candidate for profilin–actin binding, however previous studies have concluded that it is not able to bind profilin [129,132]. Similarly, the proline-rich domain of SALS is most likely not involved in profilin binding as profilin does not influence the activities of the actin binding domains of SALS [143]. Alternatively, the PRD might mediate an interaction with SH3 domains to regulate subcellular localization or activity, as has been suggested for other muscle proteins [144]. Finally, Lmods contain a C-terminal extension with a WH2 domain, which is able to bind G-actin in the barbed end groove [145], and in general, it is thought to function as an actin monomer recruiting motif in numerous other actin binding proteins [146]. At first, it was thought to play an essential role in nucleation [129,130], but later it turned out to be only secondary to ABS2 [132]. Moreover, the C-terminal extension has a more pronounced role in nucleation than the WH2 domain on its own [145]. In contrast, SALS contains a tandem WH2 domain, which is assumed to exclusively mediate its interaction with F-actin and G-actin [140,143]. Aside from the PRD and the WH2 domains, SALS is proposed to be mostly unstructured, which hinders its biochemical analysis.

The other crucial question as to the mechanisms of Lmod is whether it localizes to the pointed ends in vivo, as it is a key part of the “competition model”. Originally, it was demonstrated that Lmod is localized to the H-zone of cultured rat cardiomyocytes [129], though the pointed ends were not resolved in that study. Following that, it was demonstrated that Lmod localizes at or close to the pointed end in isolated myocytes [130,131]. They also found that intensities of Tmod1 and Lmod2 often seemed to have an inverse correlation, suggesting mutually exclusive pointed end binding [130]. However, later studies showed that Lmod is not restricted to the pointed end as it is localized to a broader region within the A-band, suggesting that Lmod might bind the sides of the thin filaments [131,137,147,148,149]. Moreover, the studies demonstrating the Tmod–Lmod overlap used diffraction limited fluorescent microscopy, which was not able to provide sufficient resolution to settle this question. Similar to Lmods, SALS is mostly localized near the H-zone during flight muscle development in *Drosophila* [140] and a recent nanoscopic analysis revealed that there was less than a 2 nm difference in the average localization of Tmod and the WH2 domain containing central region of SALS [47]. This strongly suggests that SALS is localized to the pointed end where it probably interacts/competes with Tmod, which at least in the case of SALS supports the “competition model”. A similar analysis using an appropriate vertebrate model could ultimately put an end to the controversy and determine whether Lmods are pointed end binding proteins, thin filament side binding proteins, or localized to a more central part of the H-zone.

Aside from Lmods, N-WASP—another WH2 containing actin “nucleator”—has also been linked to myofibrillogenesis as a cooperating partner of nebulin [144]. It was suggested that in response to IGF1, N-WASP is targeted to the Z-disc by nebulin, where it promotes actin incorporation and thus filament formation as a hypertrophy response [144]. However, later studies were not able to reproduce this result [150,151]. Furthermore, the suggested barbed end actin incorporation would contradict the more established pointed end elongation model of thin filaments.

Cofilin is an additional factor that has been shown to regulate thin filament length in both vertebrates and *Drosophila* [152,153], and is also required for earlier muscle development in *C. elegans* [154]. Members of the ADF/cofilin family promote the severing and disassembly of actin filaments into ADP-G-actin monomers and inhibit their nucleotide exchange (ADP to ATP). In vertebrates, the muscle specific cofilin-2 is expressed in myofibrils [155,156] and localizes in the H-zone close to the pointed ends in cultured neonatal rat cardiomyocytes [152]. Loss of cofilin-2 leads to uncontrolled actin filament growth accompanied by diminished sarcomeric Tmod1 localization, which suggests that cofilin promotes actin filament disassembly, which is necessary for the precise regulation of sarcomeric thin filaments. Furthermore, mutations in the cofilin-2 gene lead to myopathies in humans [157,158]. In addition, *Drosophila* cofilin (*twinstar*) is also required for de novo sarcomere formation at the poles of myofibers during body wall muscle development [153]. Remarkably, cofilin-2 interacts with ATP-actin monomers with higher affinity when compared to other ADF/cofilin isoforms, and it is also able to disassemble ADP-Pi-F-actin and not just ADP-F-actin. These findings suggest that cofilin-2 has specifically evolved to control the length of actin filaments in sarcomeres, which are more dynamic at their pointed ends [152,159].

Cofilin most likely cooperates with CAP, which is important for normal actin organization in multiple organisms [160,161]. CAP is an adenylyl cyclase-associated protein [162] that accelerates F-actin disassembly in the presence of ADF/cofilin and catalyzes nucleotide exchange of ADP-actin monomers [163,164]. The N-terminal helical-folded domain (HFD) of CAP is able to bind the pointed ends of cofilin decorated thin filaments and can efficiently accelerate their depolymerization rate [165,166,167]. Following the monomer dissociation, the N-terminal WH2 and CARP domains can interact with the G-actin molecules and promote their nucleotide exchange and the dissociation of cofilin [168]. In vertebrates, CAP2 is expressed in the muscles and has been found to localize to the H-zone [169,170], and a recent nanoscopic analysis revealed that it colocalizes with Tmod on the pointed end in chick embryonic cardiomyocytes [171]. Loss of function and overexpression studies suggest that CAP2 does not alter the length of thin filaments, however, it is involved in the maintenance of the G/F-actin balance and is required for α-actin isoform exchange during myofibril differentiation [171,172]. CAP2 deletion in mice leads to sudden cardiac death due to conduction abnormalities and dilated cardiomyopathy [173,174], and also causes delayed myofibril differentiation [172]. Similarly, CAP2 mutations in humans lead to supraventricular tachycardia and severe dilated cardiomyopathy [175].

To conclude, clarification of the in vivo molecular activities of Lmod seems crucial to fully understand how thin filaments elongate. Determining the precise localization of Lmod with respect to the pointed ends using fluorescent nanoscopic methods could offer a significant step forward. Importantly, the power of such an approach has been demonstrated recently on *Drosophila* flight muscles using single-molecule localization microscopy combined with structure averaging [47]. An alternative or a complementary solution would be to resolve the native structure of full-length Lmod in complex with F-actin and other associated regulatory proteins using cryo-EM.

## 5. Thin Filament Length Determination

The length of the thin filaments often varies between the different muscle types and across species, but thin filament length within a sarcomere is largely uniform [176]. While many factors involved in the assembly and elongation of thin filaments have already been identified, their presumed molecular mechanisms do not necessarily explain how the sarcomeric actin filament length might be specified. To account for thin filament uniformity, several models have been proposed including the molecular ruler hypothesis and the scaffold model.

### 5.1. Nebulin Ruler Hypothesis

Nebulin is a giant (600–900 kDa), modular actin-binding protein that has long been thought to define thin filament length by acting as a static molecular ruler [177,178,179,180,181,182,183]. Nebulin mutations are associated with nemaline myopathy in humans, which is characterized by muscle weakness and nemaline bodies [184]. In addition, the nebulin gene is connected to core-rod myopathy [185] and distal myopathies [186], therefore understanding its molecular function is of particular importance.

**Figure 4:**
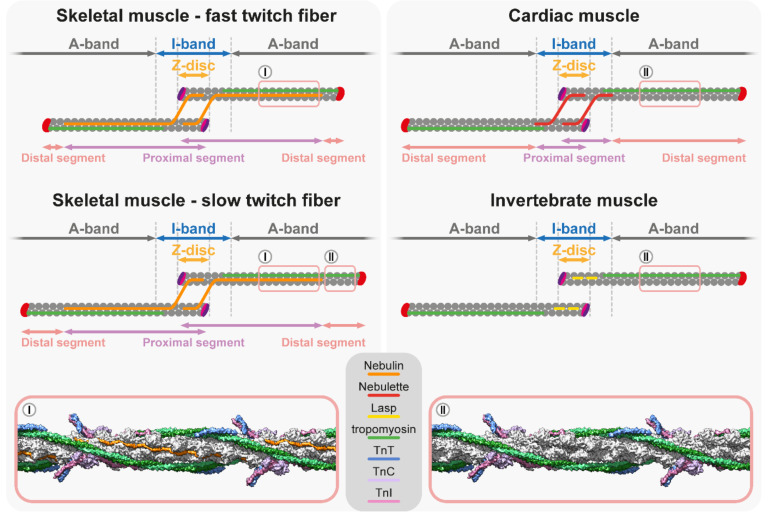
**Length regulation and the two-segment model of thin filaments.** Schematics at the top depict the two-segment model: in muscles with short distal thin filament segments (fast twitch), the length of nebulin determines the length of the thin filament by interacting with and stabilizing every actin monomer in the proximal segment. In muscles with long distal thin filament segments (slow twitch) and in cardiac muscles, the thin filament length is primarily mediated by the interplay between Tmod and Lmod at the pointed end and the role of nebulin and nebulette is only secondary. Similarly, in invertebrate (*Drosophila*) muscles, thin filament length is thought to be regulated by the interplay between Tmod and SALS, and the nebulin repeat protein Lasp is only secondary. Note that Lasp is likely to also be present in the A-band, however, in the flight muscle, it is only detected in the Z-disc. The molecular models at the bottom demonstrate that nebulin (colored in orange) is present in the grooves between the two strands of the F-actin helix in the A-band region of thin filaments [196]; PDB: 7QIM.

The ruler hypothesis advocates that the Z-disc anchored nebulin dictates thin filament length as a template by binding along each thin filament in an extended conformation with nebulin C-term in the Z-disc and N-term in the H-zone. The hypothesis also proposed that shorter thin filaments would remain dynamic until they reach the N-terminus of nebulin, which would then mediate an interaction with Tmod to cap and terminate the elongation of the thin filaments. Numerous experimental observations support this notion: (i) Nebulin was proposed to be around 1 µm long, which approximately corresponds to the length of thin filaments [180,187,188]. (ii) The length of nebulin differs in distinct muscles due to alternative splicing and its molecular weight correlates with the length of the thin filaments [180,183,189,190,191]. Nebulin contains actin binding single repeats [183], which are organized into super-repeats with each spanning one axial thin filament repeat [192]. In human skeletal muscles, the number of super-repeats in nebulin is between 22 and 29 [183,190,191]. (iii) Nebulin runs along the thin filaments while its C-terminus is anchored to the Z-disc [193] and its N-terminus is located in the vicinity of the pointed ends [182], and in vitro, the N-terminal nebulin modules (M1M2M3) are able to bind Tmod [194]. (iv) The nebulin to thin filament ratio is stoichiometric in vertebrate skeletal muscles, as two elongated nebulin molecules bind along one actin filament [177,195,196]. Accordingly, the absence of nebulin was expected to lead to unregulated thin filament lengths. However, key experiments with nebulin deficient mice showed that although the thin filaments in 1-day old animals were moderately shorter (~15–25%) compared to the control, they were initially uniform in length, and it occurred only later in 10-day old animals when they became non-uniform [197,198,199]. These experiments suggested that nebulin is somehow indeed involved in the regulation of thin filament length, but most likely not as a strict molecular ruler. Furthermore, the deteriorative thin filament uniformity suggested that nebulin is more important in the maintenance of thin filaments during muscle use than in the initial assembly and elongation of thin filaments during myogenesis. Later, it was also revealed that the pointed end of the thin filaments is typically 0.1–0.3 μm beyond the N-terminus of nebulin [4]. Additionally, another study demonstrated that thin filaments can extend beyond the end of nebulin in skeletal myocytes when the endogenous nebulin was replaced with an external ‘mini-nebulin’ molecule [200]. In light of these findings, the nebulin ruler model was no longer favored, and instead, the ‘two-segment model’ was proposed to explain the thin filament length specification and the role of nebulin [201]. The two-segment model presents the thin filaments as two-part structures: the first segment (proximal segment), starting at the Z-disc, is a nebulin-coated core region with a constant length (~0.95 µm), and the second segment next to the H-zone is a nebulin free (distal segment) region with variable length (Figure 4). The two-segment model proposed that nebulin helps to maintain the structure of the thin filament array and ensures the minimal length of the core thin filaments. However, the thin filament length is determined by the length of the distal segment, which is fine-tuned by actin dynamics at the pointed end [201]. Loss of function analysis revealed that nebulin also has non-ruler functions. These include the stabilization of the Tm and actin interactions and stabilizing actin filaments through the binding of multiple nebulin repeats along the I-band and the regulation of the stiffness of thin filaments, thereby modulating force generation [200,201,202].

Taken together, some of the above observations clearly supported the molecular ruler hypothesis, but many others have argued against it. A recent study offered a solution to this discrepancy by demonstrating that in some muscle types, nebulin indeed functions as a molecular ruler, while in others, the pointed end dynamics is the primary length regulatory mechanism, and nebulin only plays an auxiliary role as the ‘two-segment model’ suggests [203]. This study revealed that in the fast contracting mouse EDL muscle, thin filament length closely followed the length of nebulin in models where the nebulin molecule was either shortened or lengthened by inserting or deleting three nebulin super-repeats. It is important to note that the filaments still protruded ~50 nm beyond the N-terminus of nebulin, therefore, it is unlikely that pointed end capping is directed by nebulin, as suggested by the original nebulin ruler hypothesis [203]. Interestingly, in the slow contracting diaphragm and soleus muscles where the thin filament distal segments protrude far beyond the N-terminus of nebulin, the effect of the altered nebulins on the filament length was attenuated, and instead, the length was primarily mediated by the interplay between Tmod1 and Lmod2 [203]. The structure of nebulin has been recently determined by cryo-electron tomography at the near-atomic resolution from intact mouse myofibrils [196]. This structural analysis confirms that nebulin repeats bind actin with 1:1 stoichiometry and reveals that nebulin repeats stabilize actin filaments by interacting with all three neighboring actin subunits through their SDxxYK motif (Figure 4), which further strengthen their function as a molecular ruler [196].

Nebulin like simple repeats are present in other members of the nebulin protein family: N-RAP [204]; Lasp-1 [205]; Lasp-2 [206]; p80/85 [207], and Nebulette [208]. In vertebrate cardiac muscles, nebulin is only expressed in a small, sub-stoichiometric concentration (~0.2% compared to skeletal muscle) [190,209,210], and presumably the smaller nebulette is the major cardiac muscle specific Nebulin-like protein. Interfering with the function of nebulette by overexpressing a dominant-negative form in cardiomyocyte cultures shortens the thin filaments, which demonstrates its importance [211]. In humans, several mutations in the nebulette gene have been associated with dilated cardiomyopathy [212,213,214,215]. Nebulette and nebulin have similar N- and C-terminal arrangements, but they significantly differ in the number of central nebulin repeats [187,209], and the length of nebulette is not sufficient to fully span the thin filaments. Instead, nebulette is confined to the I-Z-I region of cardiac sarcomeres (Figure 4), where it binds and stabilizes thin filaments, and also plays important roles in early cardiac development [181,211,216,217]. In addition, it participates in the Z-disc alignment and the determination of the Z-disc width in mice [151,218]. In *Drosophila*, Lasp is the sole member of the nebulin family and it contains only two nebulin repeats. Despite being much shorter than nebulin, Lasp appears sufficient to fine-tune the thin filament length as in its absence, the thin filaments are ~12% shorter in the flight muscle sarcomeres [219]. Moreover, by interacting with titin and α-actinin, it controls the I-band architecture, and by interacting with both actin and myosin, it sets the proper spacing of the myofilaments [219]. Thus, shorter members of the nebulin protein family clearly play important roles in the regulation of thin filament length in various species and muscle tissues. However, instead of acting as molecular rulers, they are involved in the maintenance and stabilization of thin filaments and the fine-tuning of the myofilament architecture.

### 5.2. Titin/Myosin Scaffold Model

In contrast to the ruler model, the key mechanism of the scaffold model is the alignment of the opposite ends of the myofilaments at a particular distance from each other, without a player directly regulating the number of monomers within the thin filaments. Titin is the largest known protein that functions as a molecular spring, providing elastic properties for muscles [220,221,222,223]. Because titin is more than 1-micron long and spans from the Z-disc to the M-line, it is an excellent candidate to establish the proper spacing of the Z-discs, and consequently, to determine the length of the thin and thick filaments [221,224,225,226,227,228]. The gigantic titin protein can be separated into a Z-disc binding, a flexible I-band, a rigid A-band, and an M-line region (for a recent review see: [228]). The N-terminal Z-disc binding region associates with actin, α-actinin, and the Z-disc LIM proteins, and have been proposed to be essential to stabilize the Z-discs during myogenesis [229,230]. The I-band region contains elastic Ig domains and a unique flexible PEVK region and acts as a molecular spring [231,232,233]. The rigid A-band region contains Ig and Fn domains that form a 7-domain pattern and an 11-domain pattern in the D-zone and in the C-zone, respectively. The C-zone associated super repeats are able to bind to myosin and MyBP-C [221,234,235]. Finally, the C-terminal M-line region is directly connected to the M-line through an interaction with myomesin [236,237].

The hypothesis that titin determines the length of the thick filaments was based on the finding that it contains super-repeats that span the size (~43 nm) of the myosin helical repeats of the thick filaments [221]. This theory has recently received strong support by using a mouse model in which the first two super-repeat regions of the C-zone were missing from titin [238]. Both cardiac and skeletal muscles showed a reduced thick filament length, while the C- and N-terminal ends of the protein were at the M-line and Z-disc, respectively [238]. The actual reduction in the thick filament length (~170 nm) coincided with the length of the deleted regions (2 ∗ 43 nm at both sides of the thick filaments) [238]. Therefore, regarding the thick filaments, titin seems to act as a molecular ruler or template as it directly regulates their length with the number of super-repeats in its A-band region. Although the idea that the myosin/titin scaffold also regulates the thin filament length and determines its uniformity seems appealing, convincing experimental evidence is missing from the current literature. The size of titin in different muscles displays a great variability due to the use of distinct splice variants [239,240] that primarily differ in their flexible region. Theoretically, such a change is suitable to alter thin filament length without affecting the length of the thick filaments. Accordingly, it was demonstrated that the size of titin correlates with the thin filament length in rabbit skeletal muscles [4]. However, this correlation does not hold true in rat skeletal muscles [241], and the thin filament length is not significantly increased in mutant rats expressing an enlarged but functional titin splice variant [241,242]. Additionally, a mouse model [243] based on the expression of an enlarged titin molecule also demonstrated that the thin filament length was independent of the size of titin in cardiac sarcomeres [116]. Another argument against titin being a universal length coordinating molecule is that in many lower species such as in *Drosophila* and *C. elegans*, the titin homologs are much shorter than in vertebrates [48,244] and do not span the whole sarcomere, however, the thin filaments in these animals are still uniform and their length is tightly regulated. As these titin versions lack the A-band region, they are certainly not able to regulate the length of thick filaments in a manner that has been demonstrated in vertebrates.

While the significance of invertebrate titins in thin filament length regulation has not been fully explored, several lines of evidence suggest that the length of the myosin thick filaments plays an important role. In *Drosophila*, during flight muscle myofibrillogenesis, both filaments elongate by the same increment [245] and in the absence of myosin, thin filaments are disorganized and fail to achieve their appropriate length, as suggested by irregular Z-disc spacing [246,247]. The analysis of flightin also revealed a linkage between the length of the myofilaments. Flightin is a thick filament associated protein [248,249] that plays an important role in setting the length of the thick filaments and in maintaining their stability in *Drosophila* flight muscles [250]. In the absence of flightin, the sarcomeres of young adults undergo rapid degeneration, presumably due to the inability to withstand the forces related to the contractile activity [251,252]. However, it was possible to show that in the developing flight muscles of *flightin* null mutant animals, the thick filaments and the sarcomeres were ~25% longer than normal, while their basic sarcomere organization looked normal [250,251]. Interestingly, the thin filaments were also uniformly longer as they extended to the M-line in a similar manner as the wild type. Therefore, in the flight muscles of *Drosophila*, the thin filament length appears to be closely coordinated with that of the thick filament length. In vertebrate muscles, the length of the thick filaments is nearly constant, hence such a direct coordination is unlikely to exist, though it is plausible to assume that the elongation of the thin and thick filaments is highly synchronized with each other.

## 6. Concluding Remarks

The sarcomeric thin filament length is precisely regulated to ensure proper contraction efficiency, a key characteristic of each muscle. Mutations affecting the thin filament components, presented in the previous sections, lead to structural alterations that are the molecular basis for several debilitating muscle diseases. Therefore, understanding the mechanisms of thin filament elongation in detail would not only reveal a basic biological phenomenon, but would also help to design novel strategies to combat devastating human skeletal and cardiac muscle diseases. An important step toward this goal is to identify all proteins contributing to the regulation of thin filament length. Although the last couple of decades have been very successful in this regard, yielding a growing number of such factors, cues from non-muscle cells—where actin turnover is more thoroughly studied—suggest that additional factors might be involved. This notion is also supported by the fact that many actin associated factors have muscle specific isoforms that might be tailored to the specific needs of the sarcomeric actin filament arrays. Beyond some of the players, we are lacking in answers for a good number of mechanistic questions. These include the mechanisms of initial actin assembly, the mechanisms of pointed end elongation, the mechanisms of the radial growth of the arrays via peripheral actin assembly, the coordination of thin and thick filament growth, and a unifying theory about thin filament length regulation. To address these unresolved issues, it will be necessary to continue to combine the conventional and advanced approaches of genetics with that of newer methods such as cryo-EM, in situ cryo-ET, and fluorescent nanoscopy. Genetics and genomics will be pivotal to identifying the missing factors, whereas the application of genome editing will allow for the generation of specific mutations suitable for the surgical dissection of the often enormously huge muscle proteins. We predict that progress in our molecular level understanding will greatly depend on the application of advanced structural biology methods that might help to collect nearly as deep insights into the Z-disc/I-band and M-line/H-zone structure as our current comprehension of the sarcomeric actin–myosin interactions. One strength of the muscle research field is the traditionally broad spectra of investigations involving numerous different model systems. This is clearly in harmony with the remarkable versatility of muscle types across both evolution and within complex organisms. Thus, continuing to gather information from many different muscle models is very likely to help not only in appreciating the diversity in thin filament formation, but would also be extremely valuable in highlighting the different strategies that individual species employ to regulate thin filament dynamics. On the other hand, some of our current, apparently contradicting, mechanistic models were compiled from data derived from several different model systems. Therefore, it also appears timely to aim for a higher level comprehensive analysis of a limited number of the key model systems, equally suitable for genetic, molecular, and structural studies. We expect that combining the analysis of both classical and non-classical model systems will greatly help in better understanding the major mechanisms of thin filament regulation, together with some other critical aspects of muscle development including the separation of muscle type specific features versus the general characteristics of myofibrillogenesis.

## References

[1] Huxley A.F., Niedergerke R. (1954). Structural changes in muscle during contraction; interference microscopy of living muscle fibres. Nature.

[2] Edman K.A. (1966). The relation between sarcomere length and active tension in isolated semitendinosus fibres of the frog. J. Physiol..

[3] Gordon A.M., Huxley A.F., Julian F.J. (1966). The variation in isometric tension with sarcomere length in vertebrate muscle fibres. J. Physiol..

[4] Castillo A., Nowak R., Littlefield K.P., Fowler V.M., Littlefield R.S. (2009). A nebulin ruler does not dictate thin filament lengths. Biophys. J..

[5] Gokhin D.S., Kim N.E., Lewis S.A., Hoenecke H.R., D’Lima D.D., Fowler V.M. (2011). Thin-filament length correlates with fiber type in human skeletal muscle. Am. J. Physiol. Cell Physiol..

[6] Gokhin D.S., Lewis R.A., McKeown C.R., Nowak R.B., Kim N.E., Littlefield R.S., Lieber R.L., Fowler V.M. (2010). Tropomodulin isoforms regulate thin filament pointed-end capping and skeletal muscle physiology. J. Cell Biol..

[7] Granzier H.L., Akster H.A., Ter Keurs H.E. (1991). Effect of thin filament length on the force-sarcomere length relation of skeletal muscle. Am. J. Physiol. Cell Physiol..

[8] Ono S. (2014). Regulation of structure and function of sarcomeric actin filaments in striated muscle of the nematode Caenorhabditis elegans. Anat. Rec..

[9] Gieseler K., Qadota H., Benian G.M. (2017). Development, Structure, and Maintenance of C. Elegans Body Wall Muscle. WormBook Online Rev. C. Elegans Biol..

[10] Prill K., Dawson J.F. (2020). Assembly and Maintenance of Sarcomere Thin Filaments and Associated Diseases. Int. J. Mol. Sci..

[11] De Winter J.M., Ottenheijm C.A.C. (2017). Sarcomere Dysfunction in Nemaline Myopathy. J. Neuromuscul. Dis..

[12] Laitila J., Wallgren-Pettersson C. (2021). Recent advances in nemaline myopathy. Neuromuscul. Disord. NMD.

[13] Whitby F.G., Phillips G.N. (2000). Crystal structure of tropomyosin at 7 Angstroms resolution. Proteins.

[14] Takeda S., Yamashita A., Maeda K., Maéda Y. (2003). Structure of the core domain of human cardiac troponin in the Ca(2+)-saturated form. Nature.

[15] Vinogradova M.V., Stone D.B., Malanina G.G., Karatzaferi C., Cooke R., Mendelson R.A., Fletterick R.J. (2005). Ca(2+)-regulated structural changes in troponin. Proc. Natl. Acad. Sci. USA.

[16] Fujii T., Iwane A.H., Yanagida T., Namba K. (2010). Direct visualization of secondary structures of F-actin by electron cryomicroscopy. Nature.

[17] Lehman W., Craig R., Vibert P. (1994). Ca2+-induced tropomyosin movement in Limulus thin filaments revealed by three-dimensional reconstruction. Nature.

[18] Xu C., Craig R., Tobacman L., Horowitz R., Lehman W. (1999). Tropomyosin Positions in Regulated Thin Filaments Revealed by Cryoelectron Microscopy. Biophys. J..

[19] Narita A., Yasunaga T., Ishikawa T., Mayanagi K., Wakabayashi T. (2001). Ca(2+)-induced switching of troponin and tropomyosin on actin filaments as revealed by electron cryo-microscopy. J. Mol. Biol..

[20] Oda T., Iwasa M., Aihara T., Maéda Y., Narita A. (2009). The nature of the globular- to fibrous-actin transition. Nature.

[21] Paul D.M., Squire J.M., Morris E.P. (2010). A novel approach to the structural analysis of partially decorated actin based filaments. J. Struct. Biol..

[22] Yang S., Barbu-Tudoran L., Orzechowski M., Craig R., Trinick J., White H., Lehman W. (2014). Three-dimensional organization of troponin on cardiac muscle thin filaments in the relaxed state. Biophys. J..

[23] Paul D.M., Squire J.M., Morris E.P. (2017). Relaxed and active thin filament structures; a new structural basis for the regulatory mechanism. J. Struct. Biol..

[24] Yamada Y., Namba K., Fujii T. (2020). Cardiac muscle thin filament structures reveal calcium regulatory mechanism. Nat. Commun..

[25] Risi C.M., Pepper I., Belknap B., Landim-Vieira M., White H.D., Dryden K., Pinto J.R., Chase P.B., Galkin V.E. (2021). The structure of the native cardiac thin filament at systolic Ca(2+) levels. Proc. Natl. Acad. Sci. USA.

[26] Miller A., Tregear E.T. (1972). Structure of insect fibrillar flight muscle in the presence and absence of ATP. J. Mol. Biol..

[27] Reedy M.K., Reedy M.C. (1985). Rigor crossbridge structure in tilted single filament layers and flared-X formations from insect flight muscle. J. Mol. Biol..

[28] Schmitz H., Lucaveche C., Reedy M.K., Taylor K.A. (1994). Oblique section 3-D reconstruction of relaxed insect flight muscle reveals the cross-bridge lattice in helical registration. Biophys. J..

[29] Cooper J.A. (2002). Actin Dynamics: Tropomyosin Provides Stability. Curr. Biol..

[30] Orzechowski M., Li X.E., Fischer S., Lehman W. (2014). An atomic model of the tropomyosin cable on F-actin. Biophys. J..

[31] Marston S., Zamora J.E. (2020). Troponin structure and function: A view of recent progress. J. Muscle Res. Cell Motil..

[32] Von der Ecken J., Heissler S.M., Pathan-Chhatbar S., Manstein D.J., Raunser S. (2016). Cryo-EM structure of a human cytoplasmic actomyosin complex at near-atomic resolution. Nature.

[33] Rao J.N., Madasu Y., Dominguez R. (2014). Mechanism of actin filament pointed-end capping by tropomodulin. Science.

[34] Funk J., Merino F., Schaks M., Rottner K., Raunser S., Bieling P. (2021). A barbed end interference mechanism reveals how capping protein promotes nucleation in branched actin networks. Nat. Commun..

[35] Murakami K., Stewart M., Nozawa K., Tomii K., Kudou N., Igarashi N., Shirakihara Y., Wakatsuki S., Yasunaga T., Wakabayashi T. (2008). Structural basis for tropomyosin overlap in thin (actin) filaments and the generation of a molecular swivel by troponin-T. Proc. Natl. Acad. Sci. USA.

[36] Hanson J. (1967). Axial Period of Actin Filaments. Nature.

[37] Egelman E.H., Francis N., DeRosier D.J. (1982). F-actin is a helix with a random variable twist. Nature.

[38] Narita A., Oda T., Maéda Y. (2011). Structural basis for the slow dynamics of the actin filament pointed end. EMBO J..

[39] Zsolnay V., Katkar H.H., Chou S.Z., Pollard T.D., Voth G.A. (2020). Structural basis for polarized elongation of actin filaments. Proc. Natl. Acad. Sci. USA.

[40] Bugyi B., Papp G., Hild G., Lõrinczy D., Nevalainen E.M., Lappalainen P., Somogyi B., Nyitrai M. (2006). Formins regulate actin filament flexibility through long range allosteric interactions. J. Biol. Chem..

[41] Papp G., Bugyi B., Ujfalusi Z., Barkó S., Hild G., Somogyi B., Nyitrai M. (2006). Conformational changes in actin filaments induced by formin binding to the barbed end. Biophys. J..

[42] McGough A., Pope B., Chiu W., Weeds A. (1997). Cofilin changes the twist of F-actin: Implications for actin filament dynamics and cellular function. J. Cell Biol..

[43] Galkin V.E., Orlova A., Lukoyanova N., Wriggers W., Egelman E.H. (2001). Actin depolymerizing factor stabilizes an existing state of F-actin and can change the tilt of F-actin subunits. J. Cell Biol..

[44] Sharma S., Grintsevich E.E., Phillips M.L., Reisler E., Gimzewski J.K. (2011). Atomic force microscopy reveals drebrin induced remodeling of f-actin with subnanometer resolution. Nano Lett..

[45] Burbaum L., Schneider J., Scholze S., Böttcher R.T., Baumeister W., Schwille P., Plitzko J.M., Jasnin M. (2021). Molecular-scale visualization of sarcomere contraction within native cardiomyocytes. Nat. Commun..

[46] Wang Z., Grange M., Wagner T., Kho A.L., Gautel M., Raunser S. (2021). The molecular basis for sarcomere organization in vertebrate skeletal muscle. Cell.

[47] Szikora S., Gajdos T., Novák T., Farkas D., Földi I., Lenart P., Erdélyi M., Mihály J. (2020). Nanoscopy reveals the layered organization of the sarcomeric H-zone and I-band complexes. J. Cell Biol..

[48] Schueder F., Mangeol P., Chan E.H., Rees R., Schünemann J., Jungmann R., Görlich D., Schnorrer F. (2022). Nanobodies combined with DNA-PAINT super-resolution reveal a staggered titin nano-architecture in flight muscles. bioRxiv.

[49] Sigal Y.M., Zhou R., Zhuang X. (2018). Visualizing and discovering cellular structures with super-resolution microscopy. Science.

[50] Szikora S., Görög P., Kozma C., Mihály J. (2021). Drosophila Models Rediscovered with Super-Resolution Microscopy. Cells.

[51] Carlier M.F., Pantaloni D. (2007). Control of actin assembly dynamics in cell motility. J. Biol. Chem..

[52] Pollard T.D., Borisy G.G. (2003). Cellular motility driven by assembly and disassembly of actin filaments. Cell.

[53] Schutt C.E., Myslik J.C., Rozycki M.D., Goonesekere N.C.W., Lindberg U. (1993). The structure of crystalline profilin–β-actin. Nature.

[54] Jumper J., Evans R., Pritzel A., Green T., Figurnov M., Ronneberger O., Tunyasuvunakool K., Bates R., Žídek A., Potapenko A. (2021). Highly accurate protein structure prediction with AlphaFold. Nature.

[55] Varadi M., Anyango S., Deshpande M., Nair S., Natassia C., Yordanova G., Yuan D., Stroe O., Wood G., Laydon A. (2022). AlphaFold Protein Structure Database: Massively expanding the structural coverage of protein-sequence space with high-accuracy models. Nucleic Acids Res..

[56] Yamashita M., Higashi T., Suetsugu S., Sato Y., Ikeda T., Shirakawa R., Kita T., Takenawa T., Horiuchi H., Fukai S. (2007). Crystal structure of human DAAM1 formin homology 2 domain. Genes Cells.

[57] Kursula P., Kursula I., Massimi M., Song Y.H., Downer J., Stanley W.A., Witke W., Wilmanns M. (2008). High-resolution structural analysis of mammalian profilin 2a complex formation with two physiological ligands: The formin homology 1 domain of mDia1 and the proline-rich domain of VASP. J. Mol. Biol..

[58] Otomo T., Tomchick D.R., Otomo C., Panchal S.C., Machius M., Rosen M.K. (2005). Structural basis of actin filament nucleation and processive capping by a formin homology 2 domain. Nature.

[59] Pollard T.D., Blanchoin L., Mullins R.D. (2000). Molecular mechanisms controlling actin filament dynamics in nonmuscle cells. Annu. Rev. Biophys. Biomol. Struct..

[60] Funk J., Merino F., Venkova L., Heydenreich L., Kierfeld J., Vargas P., Raunser S., Piel M., Bieling P. (2019). Profilin and formin constitute a pacemaker system for robust actin filament growth. eLife.

[61] Shimizu N., Obinata T. (1980). Presence of Three Actin Types in Skeletal Muscle of Chick Embryos. Dev. Growth Differ..

[62] Shimizu N., Obinata T. (1986). Actin Concentration and Monomer-Polymer Ratio in Developing Chicken Skeletal Muscle 1. J. Biochem..

[63] Safer D., Nachmias V.T. (1994). Beta thymosins as actin binding peptides. BioEssays News Rev. Mol. Cell. Dev. Biol..

[64] De La Cruz E.M., Ostap E.M., Brundage R.A., Reddy K.S., Sweeney H.L., Safer D. (2000). Thymosin-β4 Changes the Conformation and Dynamics of Actin Monomers. Biophys. J..

[65] Babcock G., Rubenstein P.A. (1993). Control of profilin and actin expression in muscle and nonmuscle cells. Cell Motil. Cytoskelet..

[66] Nagaoka R., Minami N., Hayakawa K., Abe H., Obinata T. (1996). Quantitative analysis of low molecular weight G-actin-binding proteins, cofilin, ADF and profilin, expressed in developing and degenerating chicken skeletal muscles. J. Muscle Res. Cell Motil..

[67] Kooij V., Viswanathan M.C., Lee D.I., Rainer P.P., Schmidt W., Kronert W.A., Harding S.E., Kass D.A., Bernstein S.I., Van Eyk J.E. (2016). Profilin modulates sarcomeric organization and mediates cardiomyocyte hypertrophy. Cardiovasc. Res..

[68] Dominguez R. (2016). The WH2 Domain and Actin Nucleation: Necessary but Insufficient. Trends Biochem. Sci..

[69] Higgs H.N., Peterson K.J. (2005). Phylogenetic analysis of the formin homology 2 domain. Mol. Biol. Cell.

[70] Pring M., Evangelista M., Boone C., Yang C., Zigmond S.H. (2003). Mechanism of formin-induced nucleation of actin filaments. Biochemistry.

[71] Goode B.L., Eck M.J. (2007). Mechanism and function of formins in the control of actin assembly. Annu. Rev. Biochem..

[72] Paul A.S., Pollard T.D. (2009). Review of the mechanism of processive actin filament elongation by formins. Cell Motil. Cytoskelet..

[73] Kovar D.R., Kuhn J.R., Tichy A.L., Pollard T.D. (2003). The fission yeast cytokinesis formin Cdc12p is a barbed end actin filament capping protein gated by profilin. J. Cell Biol..

[74] Zigmond S.H., Evangelista M., Boone C., Yang C., Dar A.C., Sicheri F., Forkey J., Pring M. (2003). Formin leaky cap allows elongation in the presence of tight capping proteins. Curr. Biol. CB.

[75] Moseley J.B., Sagot I., Manning A.L., Xu Y., Eck M.J., Pellman D., Goode B.L. (2003). A Conserved Mechanism for Bni1- and mDia1-induced Actin Assembly and Dual Regulation of Bni1 by Bud6 and Profilin. Mol. Biol. Cell.

[76] Gutsche-Perelroizen I., Lepault J., Ott A., Carlier M.F. (1999). Filament assembly from profilin-actin. J. Biol. Chem..

[77] Kinosian H., Selden L., Gershman L., Estes J. (2002). Actin Filament Barbed End Elongation with Nonmuscle MgATP−Actin and MgADP−Actin in the Presence of Profilin. Biochemistry.

[78] Pollard T.D., Cooper J.A. (1984). Quantitative analysis of the effect of Acanthamoeba profilin on actin filament nucleation and elongation. Biochemistry.

[79] Pring M., Weber A., Bubb M.R. (1992). Profilin-actin complexes directly elongate actin filaments at the barbed end. Biochemistry.

[80] Bartolini F., Gundersen G.G. (2010). Formins and microtubules. Biochim. Biophys. Acta.

[81] Bartolini F., Moseley J.B., Schmoranzer J., Cassimeris L., Goode B.L., Gundersen G.G. (2008). The formin mDia2 stabilizes microtubules independently of its actin nucleation activity. J. Cell Biol..

[82] Gaillard J., Ramabhadran V., Neumanne E., Gurel P., Blanchoin L., Vantard M., Higgs H.N. (2011). Differential interactions of the formins INF2, mDia1, and mDia2 with microtubules. Mol. Biol. Cell.

[83] Harris E.S., Higgs H.N. (2006). Biochemical Analysis of Mammalian Formin Effects on Actin Dynamics. Methods in Enzymology.

[84] Wen Y., Eng C.H., Schmoranzer J., Cabrera-Poch N., Morris E.J., Chen M., Wallar B.J., Alberts A.S., Gundersen G.G. (2004). EB1 and APC bind to mDia to stabilize microtubules downstream of Rho and promote cell migration. Nat. Cell Biol..

[85] Vig A.T., Földi I., Szikora S., Migh E., Gombos R., Tóth M., Huber T., Pintér R., Talián G.C., Mihály J. (2017). The activities of the C-terminal regions of the formin protein disheveled-associated activator of morphogenesis (DAAM) in actin dynamics. J. Biol. Chem..

[86] Szikora S., Földi I., Tóth K., Migh E., Vig A., Bugyi B., Maléth J., Hegyi P., Kaltenecker P., Sanchez-Soriano N. (2017). The formin DAAM is required for coordination of the actin and microtubule cytoskeleton in axonal growth cones. J. Cell Sci..

[87] Foldi I., Szikora S., Mihály J. (2017). Formin’ bridges between microtubules and actin filaments in axonal growth cones. Neural Regen. Res..

[88] Rosado M., Barber C.F., Berciu C., Feldman S., Birren S.J., Nicastro D., Goode B.L. (2014). Critical roles for multiple formins during cardiac myofibril development and repair. Mol. Biol. Cell.

[89] Spletter M.L., Barz C., Yeroslaviz A., Zhang X., Lemke S.B., Bonnard A., Brunner E., Cardone G., Basler K., Habermann B.H. (2018). A transcriptomics resource reveals a transcriptional transition during ordered sarcomere morphogenesis in flight muscle. eLife.

[90] Al Haj A., Mazur A.J., Radaszkiewicz K., Radaszkiewicz T., Makowiecka A., Stopschinski B.E., Schönichen A., Geyer M., Mannherz H.G. (2015). Distribution of formins in cardiac muscle: FHOD1 is a component of intercalated discs and costameres. Eur. J. Cell Biol..

[91] Dwyer J., Pluess M., Iskratsch T., Dos Remedios C.G., Ehler E. (2014). The formin FHOD1 in cardiomyocytes. Anat. Rec..

[92] Kanaya H., Takeya R., Takeuchi K., Watanabe N., Jing N., Sumimoto H. (2005). Fhos2, a novel formin-related actin-organizing protein, probably associates with the nestin intermediate filament. Genes Cells.

[93] Taniguchi K., Takeya R., Suetsugu S., Kan O.M., Narusawa M., Shiose A., Tominaga R., Sumimoto H. (2009). Mammalian formin fhod3 regulates actin assembly and sarcomere organization in striated muscles. J. Biol. Chem..

[94] Iskratsch T., Lange S., Dwyer J., Kho A.L., dos Remedios C., Ehler E. (2010). Formin follows function: A muscle-specific isoform of FHOD3 is regulated by CK2 phosphorylation and promotes myofibril maintenance. J. Cell Biol..

[95] Kan-o M., Takeya R., Taniguchi K., Tanoue Y., Tominaga R., Sumimoto H. (2012). Expression and Subcellular Localization of Mammalian Formin Fhod3 in the Embryonic and Adult Heart. PLoS ONE.

[96] Arimura T., Takeya R., Ishikawa T., Yamano T., Matsuo A., Tatsumi T., Nomura T., Sumimoto H., Kimura A. (2013). Dilated cardiomyopathy-associated FHOD3 variant impairs the ability to induce activation of transcription factor serum response factor. Circ. J..

[97] Wooten E.C., Hebl V.B., Wolf M.J., Greytak S.R., Orr N.M., Draper I., Calvino J.E., Kapur N.K., Maron M.S., Kullo I.J. (2013). Formin homology 2 domain containing 3 variants associated with hypertrophic cardiomyopathy. Circulation. Cardiovasc. Genet..

[98] Ochoa J.P., Sabater-Molina M., García-Pinilla J.M., Mogensen J., Restrepo-Córdoba A., Palomino-Doza J., Villacorta E., Martinez-Moreno M., Ramos-Maqueda J., Zorio E. (2018). Formin Homology 2 Domain Containing 3 (FHOD3) Is a Genetic Basis for Hypertrophic Cardiomyopathy. J. Am. Coll. Cardiol..

[99] Dos Remedios C.G., Li A., Lal S. (2018). Non-sarcomeric causes of heart failure: A Sydney Heart Bank perspective. Biophys. Rev..

[100] Ushijima T., Fujimoto N., Matsuyama S., Kan O.M., Kiyonari H., Shioi G., Kage Y., Yamasaki S., Takeya R., Sumimoto H. (2018). The actin-organizing formin protein Fhod3 is required for postnatal development and functional maintenance of the adult heart in mice. J. Biol. Chem..

[101] Shwartz A., Dhanyasi N., Schejter E.D., Shilo B.-Z. (2016). The Drosophila formin Fhos is a primary mediator of sarcomeric thin-filament array assembly. eLife.

[102] Schönichen A., Mannherz H.G., Behrmann E., Mazur A.J., Kühn S., Silván U., Schoenenberger C.-A., Fackler O.T., Raunser S., Dehmelt L. (2013). FHOD1 is a combined actin filament capping and bundling factor that selectively associates with actin arcs and stress fibers. J. Cell Sci..

[103] Patel A.A., Oztug Durer Z.A., van Loon A.P., Bremer K.V., Quinlan M.E. (2018). Drosophila and human FHOD family formin proteins nucleate actin filaments. J. Biol. Chem..

[104] Fujimoto N., Kan O.M., Ushijima T., Kage Y., Tominaga R., Sumimoto H., Takeya R. (2016). Transgenic Expression of the Formin Protein Fhod3 Selectively in the Embryonic Heart: Role of Actin-Binding Activity of Fhod3 and Its Sarcomeric Localization during Myofibrillogenesis. PLoS ONE.

[105] Matsuyama S., Kage Y., Fujimoto N., Ushijima T., Tsuruda T., Kitamura K., Shiose A., Asada Y., Sumimoto H., Takeya R. (2018). Interaction between cardiac myosin-binding protein C and formin Fhod3. Proc. Natl. Acad. Sci. USA.

[106] Li D., Hallett M.A., Zhu W., Rubart M., Liu Y., Yang Z., Chen H., Haneline L.S., Chan R.J., Schwartz R.J. (2011). Dishevelled-associated activator of morphogenesis 1 (Daam1) is required for heart morphogenesis. Development.

[107] Bao B., Zhang L., Hu H., Yin S., Liang Z. (2012). Deletion of a single-copy DAAM1 gene in congenital heart defect: A case report. BMC Med. Genet..

[108] Ajima R., Bisson J.A., Helt J.-C., Nakaya M.-A., Habas R., Tessarollo L., He X., Morrisey E.E., Yamaguchi T.P., Cohen E.D. (2015). DAAM1 and DAAM2 are co-required for myocardial maturation and sarcomere assembly. Dev. Biol..

[109] Molnár I., Migh E., Szikora S., Kalmár T., Végh A.G., Deák F., Barkó S., Bugyi B., Orfanos Z., Kovács J. (2014). DAAM Is Required for Thin Filament Formation and Sarcomerogenesis during Muscle Development in Drosophila. PLoS Genet..

[110] Higashi T., Ikeda T., Shirakawa R., Kondo H., Kawato M., Horiguchi M., Okuda T., Okawa K., Fukai S., Nureki O. (2008). Biochemical characterization of the Rho GTPase-regulated actin assembly by diaphanous-related formins, mDia1 and Daam1, in platelets. J. Biol. Chem..

[111] Barkó S., Bugyi B., Carlier M.-F., Gombos R., Matusek T., Mihály J., Nyitrai M. (2010). Characterization of the Biochemical Properties and Biological Function of the Formin Homology Domains of Drosophila DAAM*. J. Biol. Chem..

[112] Deng S., Silimon R.L., Balakrishnan M., Bothe I., Juros D., Soffar D.B., Baylies M.K. (2021). The actin polymerization factor Diaphanous and the actin severing protein Flightless I collaborate to regulate sarcomere size. Dev. Biol..

[113] Deng S., Bothe I., Baylies M.K. (2015). The Formin Diaphanous Regulates Myoblast Fusion through Actin Polymerization and Arp2/3 Regulation. PLoS Genet..

[114] Mi-Mi L., Votra S., Kemphues K., Bretscher A., Pruyne D. (2012). Z-line formins promote contractile lattice growth and maintenance in striated muscles of C. elegans. J. Cell Biol..

[115] Pappas C.T., Mayfield R.M., Henderson C., Jamilpour N., Cover C., Hernandez Z., Hutchinson K.R., Chu M., Nam K.H., Valdez J.M. (2015). Knockout of Lmod2 results in shorter thin filaments followed by dilated cardiomyopathy and juvenile lethality. Proc. Natl. Acad. Sci. USA.

[116] Kolb J., Li F., Methawasin M., Adler M., Escobar Y.-N., Nedrud J., Pappas C.T., Harris S.P., Granzier H. (2016). Thin filament length in the cardiac sarcomere varies with sarcomere length but is independent of titin and nebulin. J. Mol. Cell. Cardiol..

[117] Littlefield R., Almenar-Queralt A., Fowler V.M. (2001). Actin dynamics at pointed ends regulates thin filament length in striated muscle. Nat. Cell Biol..

[118] Mardahl-Dumesnil M., Fowler V.M. (2001). Thin filaments elongate from their pointed ends during myofibril assembly in Drosophila indirect flight muscle. J. Cell Biol..

[119] Michele D.E., Albayya F.P., Metzger J.M. (1999). Thin Filament Protein Dynamics in Fully Differentiated Adult Cardiac Myocytes: Toward A Model of Sarcomere Maintenance. J. Cell Biol..

[120] Schafer D.A., Hug C., Cooper J.A. (1995). Inhibition of CapZ during myofibrillogenesis alters assembly of actin filaments. J. Cell Biol..

[121] Weber A., Pennise C.R., Babcock G.G., Fowler V.M. (1994). Tropomodulin caps the pointed ends of actin filaments. J. Cell Biol..

[122] Yamashiro S., Gokhin D.S., Kimura S., Nowak R.B., Fowler V.M. (2012). Tropomodulins: Pointed-end capping proteins that regulate actin filament architecture in diverse cell types. Cytoskeleton.

[123] Gregorio C.C., Weber A., Bondad M., Pennise C.R., Fowler V.M. (1995). Requirement of pointed-end capping by tropomodulin to maintain actin filament length in embryonic chick cardiac myocytes. Nature.

[124] Kostyukova A.S., Choy A., Rapp B.A. (2006). Tropomodulin binds two tropomyosins: A novel model for actin filament capping. Biochemistry.

[125] Sussman M.A., Welch S., Cambon N., Klevitsky R., Hewett T.E., Price R., Witt S.A., Kimball T.R. (1998). Myofibril degeneration caused by tropomodulin overexpression leads to dilated cardiomyopathy in juvenile mice. J. Clin. Investig..

[126] Gokhin D.S., Tierney M.T., Sui Z., Sacco A., Fowler V.M. (2014). Calpain-mediated proteolysis of tropomodulin isoforms leads to thin filament elongation in dystrophic skeletal muscle. Mol. Biol. Cell.

[127] Gokhin D.S., Ochala J., Domenighetti A.A., Fowler V.M. (2015). Tropomodulin 1 directly controls thin filament length in both wild-type and tropomodulin 4-deficient skeletal muscle. Development.

[128] Fowler V.M., Dominguez R. (2017). Tropomodulins and Leiomodins: Actin Pointed End Caps and Nucleators in Muscles. Biophys. J..

[129] Chereau D., Boczkowska M., Skwarek-Maruszewska A., Fujiwara I., Hayes D.B., Rebowski G., Lappalainen P., Pollard T.D., Dominguez R. (2008). Leiomodin is an actin filament nucleator in muscle cells. Science.

[130] Tsukada T., Pappas C.T., Moroz N., Antin P.B., Kostyukova A.S., Gregorio C.C. (2010). Leiomodin-2 is an antagonist of tropomodulin-1 at the pointed end of the thin filaments in cardiac muscle. J. Cell Sci..

[131] Yuen M., Sandaradura S.A., Dowling J.J., Kostyukova A.S., Moroz N., Quinlan K.G., Lehtokari V.L., Ravenscroft G., Todd E.J., Ceyhan-Birsoy O. (2014). Leiomodin-3 dysfunction results in thin filament disorganization and nemaline myopathy. J. Clin. Investig..

[132] Boczkowska M., Rebowski G., Kremneva E., Lappalainen P., Dominguez R. (2015). How Leiomodin and Tropomodulin use a common fold for different actin assembly functions. Nat. Commun..

[133] Li S., Mo K., Tian H., Chu C., Sun S., Tian L., Ding S., Li T.-R., Wu X., Liu F. (2016). Lmod2 piggyBac mutant mice exhibit dilated cardiomyopathy. Cell Biosci..

[134] Ahrens-Nicklas R.C., Pappas C.T., Farman G.P., Mayfield R.M., Larrinaga T.M., Medne L., Ritter A., Krantz I.D., Murali C., Lin K.Y. (2019). Disruption of cardiac thin filament assembly arising from a mutation in LMOD2: A novel mechanism of neonatal dilated cardiomyopathy. Sci. Adv..

[135] Cenik B.K., Garg A., McAnally J.R., Shelton J.M., Richardson J.A., Bassel-Duby R., Olson E.N., Liu N. (2015). Severe myopathy in mice lacking the MEF2/SRF-dependent gene leiomodin-3. J. Clin. Investig..

[136] Halim D., Wilson M.P., Oliver D., Brosens E., Verheij J.B., Han Y., Nanda V., Lyu Q., Doukas M., Stoop H. (2017). Loss of LMOD1 impairs smooth muscle cytocontractility and causes megacystis microcolon intestinal hypoperistalsis syndrome in humans and mice. Proc. Natl. Acad. Sci. USA.

[137] Skwarek-Maruszewska A., Boczkowska M., Zajac A.L., Kremneva E., Svitkina T., Dominguez R., Lappalainen P. (2010). Different localizations and cellular behaviors of leiomodin and tropomodulin in mature cardiomyocyte sarcomeres. Mol. Biol. Cell.

[138] Tolkatchev D., Smith G.E., Schultz L.E., Colpan M., Helms G.L., Cort J.R., Gregorio C.C., Kostyukova A.S. (2020). Leiomodin creates a leaky cap at the pointed end of actin-thin filaments. PLoS Biol..

[139] Pappas C.T., Farman G.P., Mayfield R.M., Konhilas J.P., Gregorio C.C. (2018). Cardiac-specific knockout of Lmod2 results in a severe reduction in myofilament force production and rapid cardiac failure. J. Mol. Cell. Cardiol..

[140] Bai J., Hartwig J.H., Perrimon N. (2007). SALS, a WH2-Domain-Containing Protein, Promotes Sarcomeric Actin Filament Elongation from Pointed Ends during Drosophila Muscle Growth. Dev. Cell.

[141] Tolkatchev D., Gregorio C.C., Kostyukova A.S. (2021). The role of leiomodin in actin dynamics: A new road or a secret gate. FEBS J..

[142] Ly T., Moroz N., Pappas C.T., Novak S.M., Tolkatchev D., Wooldridge D., Mayfield R.M., Helms G., Gregorio C.C., Kostyukova A.S. (2016). The N-terminal tropomyosin- and actin-binding sites are important for leiomodin 2’s function. Mol. Biol. Cell.

[143] Tóth M.Á., Majoros A.K., Vig A.T., Migh E., Nyitrai M., Mihály J., Bugyi B. (2016). Biochemical Activities of the Wiskott-Aldrich Syndrome Homology Region 2 Domains of Sarcomere Length Short (SALS) Protein*. J. Biol. Chem..

[144] Takano K., Watanabe-Takano H., Suetsugu S., Kurita S., Tsujita K., Kimura S., Karatsu T., Takenawa T., Endo T. (2010). Nebulin and N-WASP cooperate to cause IGF-1-induced sarcomeric actin filament formation. Science.

[145] Boczkowska M., Yurtsever Z., Rebowski G., Eck M.J., Dominguez R. (2017). Crystal Structure of Leiomodin 2 in Complex with Actin: A Structural and Functional Reexamination. Biophys. J..

[146] Paunola E., Mattila P.K., Lappalainen P. (2002). WH2 domain: A small, versatile adapter for actin monomers. FEBS Lett..

[147] Conley C.A., Fritz-Six K.L., Almenar-Queralt A., Fowler V.M. (2001). Leiomodins: Larger members of the tropomodulin (Tmod) gene family. Genomics.

[148] Garg A., O’Rourke J., Long C., Doering J., Ravenscroft G., Bezprozvannaya S., Nelson B.R., Beetz N., Li L., Chen S. (2014). KLHL40 deficiency destabilizes thin filament proteins and promotes nemaline myopathy. J. Clin. Investig..

[149] Szatmári D., Bugyi B., Ujfalusi Z., Grama L., Dudás R., Nyitrai M. (2017). Cardiac leiomodin2 binds to the sides of actin filaments and regulates the ATPase activity of myosin. PLoS ONE.

[150] Li F., Barton E.R., Granzier H. (2019). Deleting nebulin’s C-terminus reveals its importance to sarcomeric structure and function and is sufficient to invoke nemaline myopathy. Hum. Mol. Genet..

[151] Yamamoto D.L., Vitiello C., Zhang J., Gokhin D.S., Castaldi A., Coulis G., Piaser F., Filomena M.C., Eggenhuizen P.J., Kunderfranco P. (2013). The nebulin SH3 domain is dispensable for normal skeletal muscle structure but is required for effective active load bearing in mouse. J. Cell Sci..

[152] Kremneva E., Makkonen M.H., Skwarek-Maruszewska A., Gateva G., Michelot A., Dominguez R., Lappalainen P. (2014). Cofilin-2 controls actin filament length in muscle sarcomeres. Dev. Cell.

[153] Balakrishnan M., Yu S.F., Chin S.M., Soffar D.B., Windner S.E., Goode B.L., Baylies M.K. (2020). Cofilin Loss in Drosophila Muscles Contributes to Muscle Weakness through Defective Sarcomerogenesis during Muscle Growth. Cell Rep..

[154] Ono K., Parast M., Alberico C., Benian G.M., Ono S. (2003). Specific requirement for two ADF/cofilin isoforms in distinct actin-dependent processes in Caenorhabditis elegans. J. Cell Sci..

[155] Nakashima K., Sato N., Nakagaki T., Abe H., Ono S., Obinata T. (2005). Two mouse cofilin isoforms, muscle-type (MCF) and non-muscle type (NMCF), interact with F-actin with different efficiencies. J. Biochem..

[156] Gurniak C.B., Chevessier F., Jokwitz M., Jönsson F., Perlas E., Richter H., Matern G., Boyl P.P., Chaponnier C., Fürst D. (2014). Severe protein aggregate myopathy in a knockout mouse model points to an essential role of cofilin2 in sarcomeric actin exchange and muscle maintenance. Eur. J. Cell Biol..

[157] Agrawal P.B., Greenleaf R.S., Tomczak K.K., Lehtokari V.L., Wallgren-Pettersson C., Wallefeld W., Laing N.G., Darras B.T., Maciver S.K., Dormitzer P.R. (2007). Nemaline myopathy with minicores caused by mutation of the CFL2 gene encoding the skeletal muscle actin-binding protein, cofilin-2. Am. J. Hum. Genet..

[158] Ockeloen C.W., Gilhuis H.J., Pfundt R., Kamsteeg E.J., Agrawal P.B., Beggs A.H., Hama-Amin A.D., Diekstra A., Knoers N.V., Lammens M. (2012). Congenital myopathy caused by a novel missense mutation in the CFL2 gene. Neuromuscul. Disord. NMD.

[159] Vartiainen M.K., Mustonen T., Mattila P.K., Ojala P.J., Thesleff I., Partanen J., Lappalainen P. (2002). The three mouse actin-depolymerizing factor/cofilins evolved to fulfill cell-type-specific requirements for actin dynamics. Mol. Biol. Cell.

[160] Nomura K., Ono K., Ono S. (2012). CAS-1, a C. elegans cyclase-associated protein, is required for sarcomeric actin assembly in striated muscle. J. Cell Sci..

[161] Effendi K., Yamazaki K., Mori T., Masugi Y., Makino S., Sakamoto M. (2013). Involvement of hepatocellular carcinoma biomarker, cyclase-associated protein 2 in zebrafish body development and cancer progression. Exp. Cell Res..

[162] Field J., Vojtek A., Ballester R., Bolger G., Colicelli J., Ferguson K., Gerst J., Kataoka T., Michaeli T., Powers S. (1990). Cloning and characterization of CAP, the S. cerevisiae gene encoding the 70 kd adenylyl cyclase-associated protein. Cell.

[163] Moriyama K., Yahara I. (2002). Human CAP1 is a key factor in the recycling of cofilin and actin for rapid actin turnover. J. Cell Sci..

[164] Chaudhry F., Little K., Talarico L., Quintero-Monzon O., Goode B.L. (2010). A central role for the WH2 domain of Srv2/CAP in recharging actin monomers to drive actin turnover in vitro and in vivo. Cytoskeleton.

[165] Kotila T., Wioland H., Enkavi G., Kogan K., Vattulainen I., Jégou A., Romet-Lemonne G., Lappalainen P. (2019). Mechanism of synergistic actin filament pointed end depolymerization by cyclase-associated protein and cofilin. Nat. Commun..

[166] Shekhar S., Chung J., Kondev J., Gelles J., Goode B.L. (2019). Synergy between Cyclase-associated protein and Cofilin accelerates actin filament depolymerization by two orders of magnitude. Nat. Commun..

[167] Purde V., Busch F., Kudryashova E., Wysocki V.H., Kudryashov D.S. (2019). Oligomerization Affects the Ability of Human Cyclase-Associated Proteins 1 and 2 to Promote Actin Severing by Cofilins. Int. J. Mol. Sci..

[168] Kotila T., Kogan K., Enkavi G., Guo S., Vattulainen I., Goode B.L., Lappalainen P. (2018). Structural basis of actin monomer re-charging by cyclase-associated protein. Nat. Commun..

[169] Bertling E., Hotulainen P., Mattila P.K., Matilainen T., Salminen M., Lappalainen P. (2004). Cyclase-associated protein 1 (CAP1) promotes cofilin-induced actin dynamics in mammalian nonmuscle cells. Mol. Biol. Cell.

[170] Peche V., Shekar S., Leichter M., Korte H., Schröder R., Schleicher M., Holak T.A., Clemen C.S., Ramanath Y.B., Pfitzer G. (2007). CAP2, cyclase-associated protein 2, is a dual compartment protein. Cell. Mol. Life Sci. CMLS.

[171] Colpan M., Iwanski J., Gregorio C.C. (2021). CAP2 is a regulator of actin pointed end dynamics and myofibrillogenesis in cardiac muscle. Commun. Biol..

[172] Kepser L.J., Damar F., De Cicco T., Chaponnier C., Prószyński T.J., Pagenstecher A., Rust M.B. (2019). CAP2 deficiency delays myofibril actin cytoskeleton differentiation and disturbs skeletal muscle architecture and function. Proc. Natl. Acad. Sci. USA.

[173] Peche V.S., Holak T.A., Burgute B.D., Kosmas K., Kale S.P., Wunderlich F.T., Elhamine F., Stehle R., Pfitzer G., Nohroudi K. (2013). Ablation of cyclase-associated protein 2 (CAP2) leads to cardiomyopathy. Cell. Mol. LifeSci. CMLS.

[174] Field J., Ye D.Z., Shinde M., Liu F., Schillinger K.J., Lu M., Wang T., Skettini M., Xiong Y., Brice A.K. (2015). CAP2 in cardiac conduction, sudden cardiac death and eye development. Sci. Rep..

[175] Aspit L., Levitas A., Etzion S., Krymko H., Slanovic L., Zarivach R., Etzion Y., Parvari R. (2019). CAP2 mutation leads to impaired actin dynamics and associates with supraventricular tachycardia and dilated cardiomyopathy. J. Med. Genet..

[176] Fowler V.M. (1996). Regulation of actin filament length in erythrocytes and striated muscle. Curr. Opin. Cell Biol..

[177] Wang K., Wright J. (1988). Architecture of the sarcomere matrix of skeletal muscle: Immunoelectron microscopic evidence that suggests a set of parallel inextensible nebulin filaments anchored at the Z line. J. Cell Biol..

[178] Maruyama K., Matsuno A., Higuchi H., Shimaoka S., Kimura S., Shimizu T. (1989). Behaviour of connectin (titin) and nebulin in skinned muscle fibres released after extreme stretch as revealed by immunoelectron microscopy. J. Muscle Res. Cell Motil..

[179] Pierobon-Bormioli S., Betto R., Salviati G. (1989). The organization of titin (connectin) and nebulin in the sarcomeres: An immunocytolocalization study. J. Muscle Res. Cell Motil..

[180] Kruger M., Wright J., Wang K. (1991). Nebulin as a length regulator of thin filaments of vertebrate skeletal muscles: Correlation of thin filament length, nebulin size, and epitope profile. J. Cell Biol..

[181] Jin J.P., Wang K. (1991). Nebulin as a giant actin-binding template protein in skeletal muscle sarcomere. Interaction of actin and cloned human nebulin fragments. FEBS Lett..

[182] Wright J., Huang Q.Q., Wang K. (1993). Nebulin is a full-length template of actin filaments in the skeletal muscle sarcomere: An immunoelectron microscopic study of its orientation and span with site-specific monoclonal antibodies. J. Muscle Res. Cell Motil..

[183] Labeit S., Kolmerer B. (1995). The complete primary structure of human nebulin and its correlation to muscle structure. J. Mol. Biol..

[184] Lehtokari V.L., Pelin K., Sandbacka M., Ranta S., Donner K., Muntoni F., Sewry C., Angelini C., Bushby K., Van den Bergh P. (2006). Identification of 45 novel mutations in the nebulin gene associated with autosomal recessive nemaline myopathy. Hum. Mutat..

[185] Romero N.B., Lehtokari V.L., Quijano-Roy S., Monnier N., Claeys K.G., Carlier R.Y., Pellegrini N., Orlikowski D., Barois A., Laing N.G. (2009). Core-rod myopathy caused by mutations in the nebulin gene. Neurology.

[186] Scoto M., Cullup T., Cirak S., Yau S., Manzur A.Y., Feng L., Jacques T.S., Anderson G., Abbs S., Sewry C. (2013). Nebulin (NEB) mutations in a childhood onset distal myopathy with rods and cores uncovered by next generation sequencing. Eur. J. Hum. Genet. EJHG.

[187] Pappas C.T., Bliss K.T., Zieseniss A., Gregorio C.C. (2011). The Nebulin family: An actin support group. Trends Cell Biol..

[188] Ottenheijm C.A.C., Granzier H., Labeit S. (2012). The sarcomeric protein nebulin: Another multifunctional giant in charge of muscle strength optimization. Front. Physiol.

[189] Labeit S., Gibson T., Lakey A., Leonard K., Zeviani M., Knight P., Wardale J., Trinick J. (1991). Evidence that nebulin is a protein-ruler in muscle thin filaments. FEBS Lett..

[190] Donner K., Sandbacka M., Lehtokari V.L., Wallgren-Pettersson C., Pelin K. (2004). Complete genomic structure of the human nebulin gene and identification of alternatively spliced transcripts. Eur. J. Hum. Genet. EJHG.

[191] Laitila J., Hanif M., Paetau A., Hujanen S., Keto J., Somervuo P., Huovinen S., Udd B., Wallgren-Pettersson C., Auvinen P. (2012). Expression of multiple nebulin isoforms in human skeletal muscle and brain. Muscle Nerve.

[192] Marttila M., Hanif M., Lemola E., Nowak K.J., Laitila J., Grönholm M., Wallgren-Pettersson C., Pelin K. (2014). Nebulin interactions with actin and tropomyosin are altered by disease-causing mutations. Skelet. Muscle.

[193] Pappas C.T., Bhattacharya N., Cooper J.A., Gregorio C.C. (2008). Nebulin interacts with CapZ and regulates thin filament architecture within the Z-disc. Mol. Biol. Cell.

[194] McElhinny A.S., Kolmerer B., Fowler V.M., Labeit S., Gregorio C.C. (2001). The N-terminal end of nebulin interacts with tropomodulin at the pointed ends of the thin filaments. J. Biol. Chem..

[195] Buck D., Hudson B.D., Ottenheijm C.A., Labeit S., Granzier H. (2010). Differential splicing of the large sarcomeric protein nebulin during skeletal muscle development. J. Struct. Biol..

[196] Wang Z., Grange M., Pospich S., Wagner T., Kho A.L., Gautel M., Raunser S. (2022). Structures from intact myofibrils reveal mechanism of thin filament regulation through nebulin. Science.

[197] Bang M.-L., Li X., Littlefield R., Bremner S., Thor A., Knowlton K.U., Lieber R.L., Chen J. (2006). Nebulin-deficient mice exhibit shorter thin filament lengths and reduced contractile function in skeletal muscle. J. Cell Biol..

[198] Witt C.C., Burkart C., Labeit D., McNabb M., Wu Y., Granzier H., Labeit S. (2006). Nebulin regulates thin filament length, contractility, and Z-disk structure in vivo. EMBO J..

[199] Ottenheijm C.A.C., Witt C.C., Stienen G.J., Labeit S., Beggs A.H., Granzier H. (2009). Thin filament length dysregulation contributes to muscle weakness in nemaline myopathy patients with nebulin deficiency. Hum. Mol. Genet..

[200] Pappas C.T., Krieg P.A., Gregorio C.C. (2010). Nebulin regulates actin filament lengths by a stabilization mechanism. J. Cell Biol..

[201] Gokhin D.S., Fowler V.M. (2013). A two-segment model for thin filament architecture in skeletal muscle. Nat. Rev. Mol. Cell Biol..

[202] Kiss B., Lee E.J., Ma W., Li F.W., Tonino P., Mijailovich S.M., Irving T.C., Granzier H.L. (2018). Nebulin stiffens the thin filament and augments cross-bridge interaction in skeletal muscle. Proc. Natl. Acad. Sci. USA.

[203] Kiss B., Gohlke J., Tonino P., Hourani Z., Kolb J., Strom J., Alekhina O., Smith J.E., Ottenheijm C., Gregorio C. (2020). Nebulin and Lmod2 are critical for specifying thin-filament length in skeletal muscle. Sci. Adv..

[204] Luo G., Zhang J.Q., Nguyen T.P., Herrera A.H., Paterson B., Horowits R. (1997). Complete cDNA sequence and tissue localization of N-RAP, a novel nebulin-related protein of striated muscle. Cell Motil. Cytoskelet..

[205] Schreiber V., Moog-Lutz C., Régnier C.H., Chenard M.-P., Boeuf H., Vonesch J.-L., Tomasetto C., Rio M.-C. (1998). Lasp-1, a Novel Type of Actin-Binding Protein Accumulating in Cell Membrane Extensions. Mol. Med..

[206] Li B., Zhuang L., Trueb B. (2004). Zyxin interacts with the SH3 domains of the cytoskeletal proteins LIM-nebulette and Lasp-1. J. Biol. Chem..

[207] Wu H., Reynolds A.B., Kanner S.B., Vines R.R., Parsons J.T. (1991). Identification and characterization of a novel cytoskeleton-associated pp60src substrate. Mol. Cell. Biol..

[208] Moncman C.L., Wang K. (1995). Nebulette: A 107 kD nebulin-like protein in cardiac muscle. Cell Motil. Cytoskelet..

[209] Bang M.L., Chen J. (2015). Roles of Nebulin Family Members in the Heart. Circ. J..

[210] Kazmierski S.T., Antin P.B., Witt C.C., Huebner N., McElhinny A.S., Labeit S., Gregorio C.C. (2003). The complete mouse nebulin gene sequence and the identification of cardiac nebulin. J. Mol. Biol..

[211] Moncman C.L., Wang K. (1999). Functional dissection of nebulette demonstrates actin binding of nebulin-like repeats and Z-line targeting of SH3 and linker domains. Cell Motil Cytoskelet..

[212] Arimura T., Nakamura T., Hiroi S., Satoh M., Takahashi M., Ohbuchi N., Ueda K., Nouchi T., Yamaguchi N., Akai J. (2000). Characterization of the human nebulette gene: A polymorphism in an actin-binding motif is associated with nonfamilial idiopathic dilated cardiomyopathy. Hum. Genet..

[213] Purevjav E., Varela J., Morgado M., Kearney D.L., Li H., Taylor M.D., Arimura T., Moncman C.L., McKenna W., Murphy R.T. (2010). Nebulette mutations are associated with dilated cardiomyopathy and endocardial fibroelastosis. J. Am. Coll. Cardiol..

[214] Maiellaro-Rafferty K., Wansapura J.P., Mendsaikhan U., Osinska H., James J.F., Taylor M.D., Robbins J., Kranias E.G., Towbin J.A., Purevjav E. (2013). Altered regional cardiac wall mechanics are associated with differential cardiomyocyte calcium handling due to nebulette mutations in preclinical inherited dilated cardiomyopathy. J. Mol. Cell. Cardiol..

[215] Perrot A., Tomasov P., Villard E., Faludi R., Melacini P., Lossie J., Lohmann N., Richard P., De Bortoli M., Angelini A. (2016). Mutations in NEBL encoding the cardiac Z-disk protein nebulette are associated with various cardiomyopathies. Arch. Med. Sci. AMS.

[216] Ogut O., Hossain M.M., Jin J.P. (2003). Interactions between nebulin-like motifs and thin filament regulatory proteins. J. Biol. Chem..

[217] Esham M., Bryan K., Milnes J., Holmes W.B., Moncman C.L. (2007). Expression of nebulette during early cardiac development. Cell Motil. Cytoskelet..

[218] Mastrototaro G., Liang X., Li X., Carullo P., Piroddi N., Tesi C., Gu Y., Dalton N.D., Peterson K.L., Poggesi C. (2015). Nebulette knockout mice have normal cardiac function, but show Z-line widening and up-regulation of cardiac stress markers. Cardiovasc. Res..

[219] Fernandes I., Schöck F. (2014). The nebulin repeat protein Lasp regulates I-band architecture and filament spacing in myofibrils. J. Cell Biol..

[220] Wang K., McClure J., Tu A. (1979). Titin: Major myofibrillar components of striated muscle. Proc. Natl. Acad. Sci. USA.

[221] Fürst D.O., Osborn M., Nave R., Weber K. (1988). The organization of titin filaments in the half-sarcomere revealed by monoclonal antibodies in immunoelectron microscopy: A map of ten nonrepetitive epitopes starting at the Z line extends close to the M line. J. Cell Biol..

[222] Trombitás K., Jin J.P., Granzier H. (1995). The mechanically active domain of titin in cardiac muscle. Circ. Res..

[223] Kellermayer M.S., Smith S.B., Granzier H.L., Bustamante C. (1997). Folding-unfolding transitions in single titin molecules characterized with laser tweezers. Science.

[224] Maruyama K., Yoshioka T., Higuchi H., Ohashi K., Kimura S., Natori R. (1985). Connectin filaments link thick filaments and Z lines in frog skeletal muscle as revealed by immunoelectron microscopy. J. Cell Biol..

[225] Whiting A., Wardale J., Trinick J. (1989). Does titin regulate the length of muscle thick filaments?. J. Mol. Biol..

[226] Bennett P.M., Gautel M. (1996). Titin Domain Patterns Correlate with the Axial Disposition of Myosin at the End of the Thick Filament. J. Mol. Biol..

[227] Bennett P., Rees M., Gautel M. (2020). The Axial Alignment of Titin on the Muscle Thick Filament Supports Its Role as a Molecular Ruler. J. Mol. Biol..

[228] Tskhovrebova L., Trinick J. (2017). Titin and Nebulin in Thick and Thin Filament Length Regulation. Subcell. Biochem..

[229] Gregorio C.C., Trombitás K., Centner T., Kolmerer B., Stier G., Kunke K., Suzuki K., Obermayr F., Herrmann B., Granzier H. (1998). The NH2 terminus of titin spans the Z-disc: Its interaction with a novel 19-kD ligand (T-cap) is required for sarcomeric integrity. J. Cell Biol..

[230] Peckham M., Young P., Gautel M. (1997). Constitutive and variable regions of Z-disk titin/connectin in myofibril formation: A dominant-negative screen. Cell Struct. Funct..

[231] Linke W.A., Ivemeyer M., Mundel P., Stockmeier M.R., Kolmerer B. (1998). Nature of PEVK-titin elasticity in skeletal muscle. Proc. Natl. Acad. Sci. USA.

[232] Opitz C.A., Kulke M., Leake M.C., Neagoe C., Hinssen H., Hajjar R.J., Linke W.A. (2003). Damped elastic recoil of the titin spring in myofibrils of human myocardium. Proc. Natl. Acad. Sci. USA.

[233] Granzier H.L., Labeit S. (2005). Titin and its associated proteins: The third myofilament system of the sarcomere. Adv. Protein Chem..

[234] Muhle-Goll C., Habeck M., Cazorla O., Nilges M., Labeit S., Granzier H. (2001). Structural and functional studies of titin’s fn3 modules reveal conserved surface patterns and binding to myosin S1—A possible role in the Frank-Starling mechanism of the heart. J. Mol. Biol..

[235] Freiburg A., Gautel M. (1996). A molecular map of the interactions between titin and myosin-binding protein C. Implications for sarcomeric assembly in familial hypertrophic cardiomyopathy. Eur. J. Biochem..

[236] Obermann W.M., Gautel M., Weber K., Fürst D.O. (1997). Molecular structure of the sarcomeric M band: Mapping of titin and myosin binding domains in myomesin and the identification of a potential regulatory phosphorylation site in myomesin. EMBO J..

[237] Kontrogianni-Konstantopoulos A., Ackermann M.A., Bowman A.L., Yap S.V., Bloch R.J. (2009). Muscle giants: Molecular scaffolds in sarcomerogenesis. Physiol. Rev..

[238] Tonino P., Kiss B., Strom J., Methawasin M., Smith J.E., Kolb J., Labeit S., Granzier H. (2017). The giant protein titin regulates the length of the striated muscle thick filament. Nat. Commun..

[239] Prado L.G., Makarenko I., Andresen C., Krüger M., Opitz C.A., Linke W.A. (2005). Isoform diversity of giant proteins in relation to passive and active contractile properties of rabbit skeletal muscles. J. Gen. Physiol..

[240] Guo W., Bharmal S.J., Esbona K., Greaser M.L. (2010). Titin diversity—Alternative splicing gone wild. J. Biomed. Biotechnol..

[241] Greaser M.L., Pleitner J.M. (2014). Titin isoform size is not correlated with thin filament length in rat skeletal muscle. Front. Physiol..

[242] Greaser M.L., Warren C.M., Esbona K., Guo W., Duan Y., Parrish A.M., Krzesinski P.R., Norman H.S., Dunning S., Fitzsimons D.P. (2008). Mutation that dramatically alters rat titin isoform expression and cardiomyocyte passive tension. J. Mol. Cell. Cardiol..

[243] Methawasin M., Hutchinson K.R., Lee E.J., Smith J.E., Saripalli C., Hidalgo C.G., Ottenheijm C.A., Granzier H. (2014). Experimentally increasing titin compliance in a novel mouse model attenuates the Frank-Starling mechanism but has a beneficial effect on diastole. Circulation.

[244] Hooper S.L., Thuma J.B. (2005). Invertebrate muscles: Muscle specific genes and proteins. Physiol. Rev..

[245] Reedy M.C., Beall C. (1993). Ultrastructure of developing flight muscle in Drosophila. I. Assembly of myofibrils. Dev. Biol..

[246] Chun M., Falkenthal S. (1988). Ifm(2)2 is a myosin heavy chain allele that disrupts myofibrillar assembly only in the indirect flight muscle of Drosophila melanogaster. J. Cell Biol..

[247] Bernstein S.I., O’Donnell P.T., Cripps R.M., Jeon K.W., Friedlander M., Jarvik J. (1993). Molecular Genetic Analysis of Muscle Development, Structure, and Function in Drosophila. International Review of Cytology.

[248] Vigoreaux J.O., Saide J.D., Valgeirsdottir K., Pardue M.L. (1993). Flightin, a novel myofibrillar protein of Drosophila stretch-activated muscles. J. Cell Biol..

[249] Ayer G., Vigoreaux J.O. (2003). Flightin is a myosin rod binding protein. Cell Biochem. Biophys..

[250] Reedy M.C., Bullard B., Vigoreaux J.O. (2000). Flightin is essential for thick filament assembly and sarcomere stability in Drosophila flight muscles. J. Cell Biol..

[251] Contompasis J.L., Nyland L.R., Maughan D.W., Vigoreaux J.O. (2010). Flightin Is Necessary for Length Determination, Structural Integrity, and Large Bending Stiffness of Insect Flight Muscle Thick Filaments. J. Mol. Biol..

[252] Gasek N.S., Nyland L.R., Vigoreaux J.O. (2016). The Contributions of the Amino and Carboxy Terminal Domains of Flightin to the Biomechanical Properties of Drosophila Flight Muscle Thick Filaments. Biology.

